# Natriuretic peptides are neuroprotective on in vitro models of PD and promote dopaminergic differentiation of hiPSCs-derived neurons via the Wnt/β-catenin signaling

**DOI:** 10.1038/s41420-021-00723-6

**Published:** 2021-11-01

**Authors:** Daniela Giovannini, Federica Andreola, Paola Spitalieri, Ewa Krystyna Krasnowska, Arianna Colini Baldeschi, Simona Rossi, Federica Sangiuolo, Mauro Cozzolino, Annalucia Serafino

**Affiliations:** 1 Institute of Translational Pharmacology—National Research Council of Italy, Rome, Italy; 2 Department of Biomedicine and Prevention, Genetic Medicine Unit, University of Rome “Tor Vergata”, Rome, Italy

**Keywords:** Target validation, Parkinson's disease

## Abstract

Over the last 20 years, the efforts to develop new therapies for Parkinson’s disease (PD) have focused not only on the improvement of symptomatic therapy for motor and non-motor symptoms but also on the discovering of the potential causes of PD, in order to develop disease-modifying treatments. The emerging role of dysregulation of the Wnt/β-catenin signaling in the onset and progression of PD, as well as of other neurodegenerative diseases (NDs), renders the targeting of this signaling an attractive therapeutic opportunity for curing this brain disorder. The natriuretic peptides (NPs) atrial natriuretic peptide (ANP), brain natriuretic peptide (BNP), and C-type natriuretic peptide (CNP), are cardiac and vascular-derived hormones also widely expressed in mammalian CNS, where they seem to participate in numerous brain functions including neural development/differentiation and neuroprotection. We recently demonstrated that ANP affects the Wnt/β-catenin pathway possibly through a Frizzled receptor-mediated mechanism and that it acts as a neuroprotective agent in in vitro models of PD by upregulating this signaling. Here we provide further evidence supporting the therapeutic potential of this class of natriuretic hormones. Specifically, we demonstrate that all the three natriuretic peptides are neuroprotective for SHSY5Y cells and primary cultures of DA neurons from mouse brain, subjected to neurotoxin insult with 6-hydroxydopamine (6-OHDA) for mimicking the neurodegeneration of PD, and these effects are associated with the activation of the Wnt/β-catenin pathway. Moreover, ANP, BNP, CNP are able to improve and accelerate the dopaminergic differentiation and maturation of hiPSCs-derived neural population obtained from two differed healthy donors, concomitantly affecting the canonical Wnt signaling. Our results support the relevance of exogenous ANP, BNP, and CNP as attractive molecules for both neuroprotection and neurorepair in PD, and more in general, in NDs for which aberrant Wnt signaling seems to be the leading pathogenetic mechanism.

## Introduction

Parkinson’s disease (PD) is the most common movement disorder and the second most frequent aging-related neurodegenerative disease (ND) after Alzheimer’s disease (AD). It is characterized by progressive degeneration of midbrain dopaminergic (mDA) neurons in the *subtantia nigra pars compacta* [1, 2], and their projections into the *striatum*, which leads to a considerable reduction in dopamine levels and to accumulation of pathological α-synuclein, the major component of Lewy bodies, considered as the most typical hallmarks of PD [3–5]. The progressive loss of dopamine input to the *striatum* underlays most of the motor symptoms associated with the disease including resting tremors, rigidity, bradykinesia, and postural instability, while non­-motor symptoms, consisting in progressive impairment of autonomic, cognitive, and mood functions, are associated to damage of other regions of the central and peripheral nervous system [2, 6, 7].

The exact molecular mechanisms underlying the onset of PD are still unknown, even if genetic and environmental factors, such as environmental pollutants and lifestyles, that lead to neuroinflammation, oxidative stress, mitochondrial dysfunction, alteration in neurotransmitter receptors [8–10], seem to be possible triggers. Currently, a disease-modifying therapy for PD does not exist, and treatments for this neurological disorder mostly consist of pharmacotherapy to restore striatal dopamine levels, that only temporarily reduce symptoms [4], and whose long-term use causes adverse events including reduced efficacy and dyskinesias. Thus, research for new treatments is mandatory and the deepening of knowledge on the molecular mechanisms triggering PD onset is crucial to discover innovative targets useful for developing more effective therapeutic strategies.

The Wnt/β-catenin signaling (or canonical Wnt signaling) is an evolutionarily conserved pathway having a crucial role in both normal embryonic development [11–14] and maintenance of adult tissue homeostasis [15–19]. In the central nervous system (CNS), the Wnt pathway regulates different aspects of neuronal functions including differentiation, synapse formation, neurogenesis, and neuroprotection [14, 20–22]. In the last decades, increasing evidence suggested that the Wnt/β-catenin signaling has a critical role in mDA neuron development [23, 24], and participates in adult neurogenesis of the hippocampus [25, 26]. Only recently Wnt ligand and other components of the pathway have been described in the midbrain of adults and PD patients [27, 28], and dysregulation of this pathway has been proposed as a new pathological mechanism leading to PD [29] and other NDs [30–34]. Hence, this signaling has been proposed as a potential therapeutic target against neurodegeneration [34, 35]. It has also been reported that appropriate levels of Wnt signaling are critical for increasing the quantity and quality of mDA neurons derived from stem cells or reprogrammed cells, with clear implications for their use in cell replacement strategies, currently under investigation as a potential PD therapy [36–38]. Indeed, since the main pathological feature of PD is neuron death or deactivation in the midbrain, a promising therapeutic strategy for curing this disease is DA neuron regeneration [37–39], by transplanting neurons differentiated from neural stem cells in vitro or, even better, by promoting the neurogenic regeneration of neural stem cells in situ through their proliferation and differentiation into functional neurons [40]. On this ground, the established role of the Wnt/β-catenin signaling in adult neurogenesis [21, 25, 26], expands the attractiveness of this pathway as a therapeutic target for PD. In intact midbrain, Wnt/β-cat signaling functions as a microenvironmental [38] sensor that balances cell survival and death [28]. In-depth, Wnt ligands, by binding to the Wnt signaling receptor Frizzled (Fzd) and the LRP5/6 co-receptors, activate the pathway (*Wnt-on*) by blocking the GSK-3β-induced phosphorylation and degradation of β-catenin at the proteasome. Stabilized β-catenin accumulates in the cytoplasm and translocates into the nucleus where, by acting as a co-activator for TCF/LEF-mediated transcription, it triggers the expression of target genes involved in neuron survival and plasticity, hence maintaining the neuron integrity [34]. Various antioxidant and anti-inflammatory molecules, as well as neuroprotective factors, are also capable to trigger the signaling [27, 28]. Conversely, Wnt antagonists, oxidative stress, inflammation, neurotoxic agents, growth factor deprivation, or aging antagonize the Wnt/β-catenin signaling in neurons. In this state of *Wnt-off*, the transcription of Wnt target genes, involved in neuron survival, is inhibited, since the β-catenin excess is rapidly phosphorylated by GSK-3β at the APC/axin/GSK-3β destruction complex and is then subjected to ubiquitin–proteasomal degradation.

**Fig. 1:**
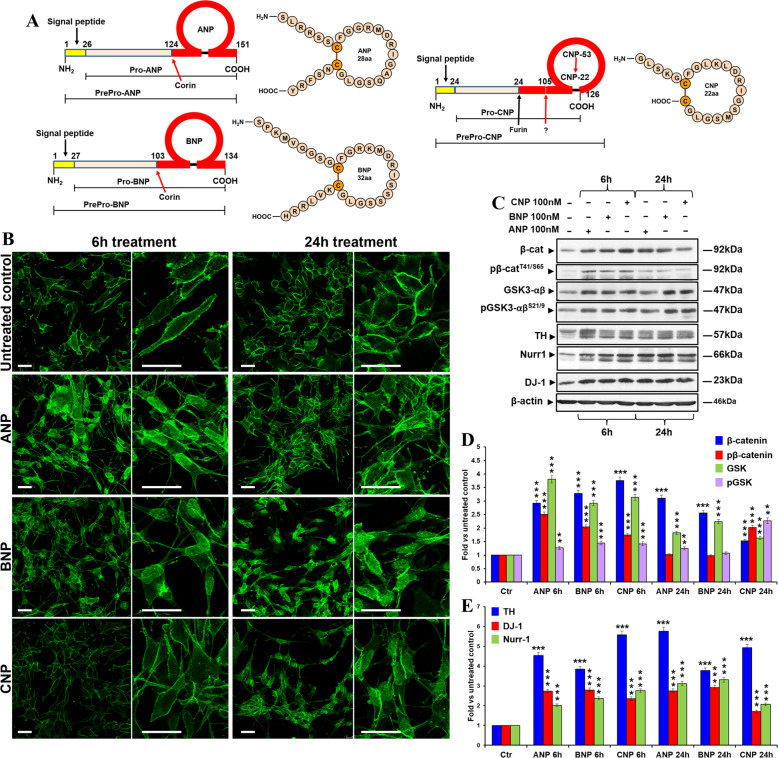
NPs induce β-catenin stabilization and nuclear translocation and affect DA neuron markers and PD-related survival factors in SHSY5Y cells. **A** Structure of the human natriuretic peptides. Pro-ANP and pro-BNP are proteolytically cleaved to the mature forms by the membrane-associated protease Corin [43], which extracellular region, that contains the structural domains essential the processing, includes the Wnt signaling receptors Fzd1 and Fzd2 [44]. Differently, the processing of pro-CNP to its mature form occurs through the action of the intracellular serine endoprotease furin [45], which generates the 53aa biologically active peptide (CNP-53), the main active form of CNP at the tissue level. CNP-53 is then further cleaved by an unknown protease to a shorter 22aa form (CNP-22), which is the dominant form in the systemic circulation [46]. The structure of the prepro-hormones for ANP, BNP, and CNP are reported on the left of each panel, while the final amino acid sequence and structure of the mature and biologically active peptides are shown on the right. The sites of cleavage operated by Corin (for ANP and BNP) and by Furin (for CNP) are also indicated. **B** Confocal microscopic images showing the intracellular distribution of β-catenin in control and NPs treated cells after 6 and 24 h of culture. Bars 25 µm. **C** WB analyses of total and phosphorylated β-catenin (pβ-cateninT41/S65), total and phosphorylated GSK-3β (pGSK-3βSer9), TH, Nurr-1, and DJ-1 levels in total cell lysates from NPs treated SHSY5Y cells, compared to the untreated control, after 6 and 24 h of treatment. **D**, **E** Densitometric analysis of total and phosphorylated β-catenin and GSK-3β (**D**), and of TH, Nurr-1, and DJ-1 (**E**) using the ImageJ processing program; values were normalized to β-actin, or vs. the total levels of each protein for the phosphorylated forms. Results are the mean ± SD from three independent experiments (*n* = 3). Significance vs. untreated relative control (one-way mANOVA + Tukey multiple comparison test): ^**^p < 0.01, ^***^p < 0.001. Additional results from the one‐way ANOVA test used to analyze the differences between groups are reported in Supplementary Data.

The natriuretic peptides (NPs) atrial NP (ANP), brain NP (BNP), and C-type NP (CNP), is cardiac and vascular-derived hormones that act in an endocrine or paracrine fashion mainly regulating the extracellular fluid volume and blood pressure [41, 42]. ANP, BNP, and CNP are synthesized as pre–pro-hormones and are proteolytically processed to form the mature peptides. The cleavage is operated, for ANP and BNP, by the Corin [43], a membrane-associated protease which includes in the extracellular region the two Fzd-like cysteine-rich domains Fzd1 and Fzd2, receptors for Wnt signaling [44], and by the intracellular serine endoprotease furin [45], for CNP (CNP-53), that is further cleaved by an unknown protease to a shorter 22aa form [46] (Fig. 1A). The three peptides share a similar structure consisting of two cysteine residues flanking a 17-residue disulfide-linked ring that is essential for their biological activity (Fig. 1A). In addition to their natriuretic effects, the three hormones seem to contribute to various brain functions [47, 48]. Indeed, NPs and their specific natriuretic receptors (NPRs) are widely expressed in mammalian CNS and a large body of evidence indicates that they could be involved in the regulation of neural development and differentiation, synaptic transmission and information processing, blood–brain barrier integrity, brain fluid homeostasis, neuroinflammation, and neuroprotection [48–50]. Given their roles in the CNS, supported by neurobiological and clinical evidence, NPs have been recently proposed as potential innovative diagnostic and therapeutic targets for some brain disorders related to a cognitive impairment such as AD [48, 51, 52]. We recently demonstrated that ANP affects the Wnt/β-catenin pathway possibly through an Fzd receptor-mediated mechanism [53, 54], and that it acts as a neuroprotective agent in in vitro models of PD by upregulating this signaling [55].

To provide further evidence supporting the therapeutic potential of this class of natriuretic hormones, in this work we verified if also BNP and CNP were able to positively affect the Wnt/β-cat signaling and to possess neuroprotective abilities in in vitro models of PD, similarly to what previously demonstrated for ANP [55]. Furthermore, owing to the reported involvement of the Wnt/β-catenin pathway in adult neurogenesis and the suggested role of NPs in the regulation of neural development and differentiation, we assessed if the three NPs were also capable of stimulating the differentiation toward the dopaminergic linage of partially committed human induced pluripotent stem cells (hiPSCs), used as a model of neural stem cells.

## Results

### BNP and CNP affect the Wnt/β-catenin signaling and increase the expression of DA neuron markers and PD-related survival factors in DA neuron-like cells

In order to assess whether BNP and CNP treatments affected the Wnt/β-catenin signaling as previously demonstrated for ANP [55], the neuroblastoma SHSY5Y cells, used as a model of DA neurons [55], were treated with the two NPs and the effects on β-catenin intracellular distribution and degradation were then analyzed after 6 and 24 h and compared with those of ANP, used as a positive control of efficacy (Fig. 1B–D). β-catenin nuclear translocation and the concomitant increase in its expression have been used as main markers of canonical Wnt pathway activation. Confocal microscopic analysis showed that all the three NPs were able to induce β-catenin translocation from cell membrane toward the cytoplasmic and nuclear compartments, even if this event occurs earlier under ANP and BNP stimulation (already after 6 h of treatment) compared to what was observed for CNP (after 24 h) (Fig. 1B). WB analysis confirmed that BNP and CNP, similarly to ANP, induce an increase of total β-catenin already after 6 h of treatment. Furthermore, between 6 and 24 h, a significant decrease in β-catenin phosphorylation at Thr41/Ser65 (pβ-catenin^T41/S65^), one of the molecular events upstream β-catenin ubiquitination and degradation at the proteasome, was recorded for ANP and BNP, but not for CNP (Fig. 1C, D). Nevertheless, all the three NPs induced an increase of GSK phosphorylation levels at Ser9 (pGSK-3β^Ser9^), the inactive form of this enzyme [56] deputed to β-catenin phosphorylation at Thr41 (Fig. 1C, D). Overall, these results indicate that, in SHSY5Y cells, all the three NPs activate the canonical Wnt signaling by stimulating β-catenin stabilization and nuclear translocation, even if with some differences in the phosphorylation kinetics of β-catenin and GSK for CNP (Fig. 1C, D). Moreover, at both times examined, highly significant increases in the expression levels of the DA neuron markers tyrosine hydroxylase (TH) and nuclear receptor-related 1 protein (Nurr1) as well as of the PD-related survival factor involved in neuroprotection DJ-1, have been recorded under treatments with all the three NPs (Fig. 1C, E).

### BNP and CNP pre-treatments prevent neurotoxin-induced toxicity in SHSY5Y cells

**Fig. 2:**
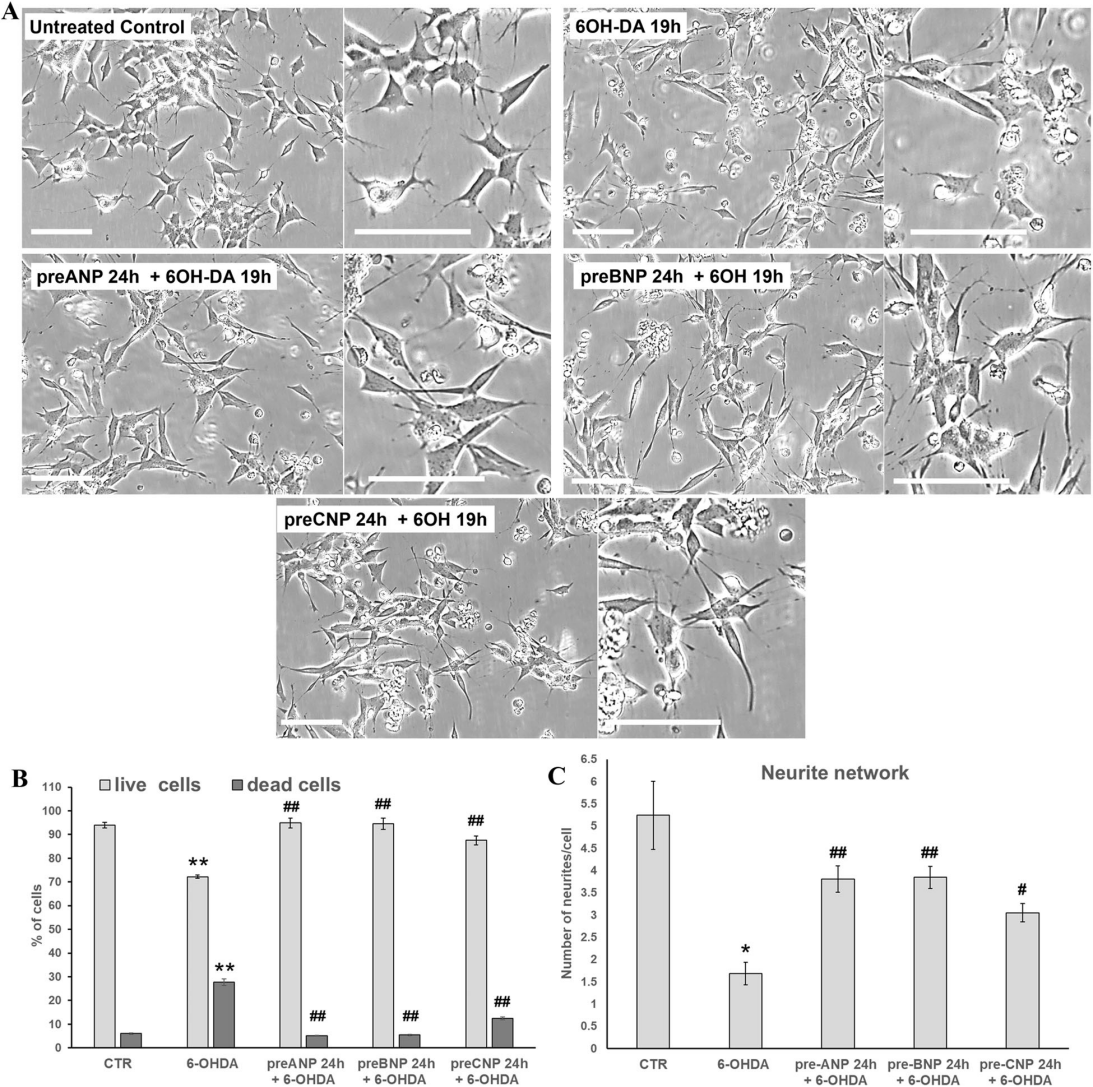
NPs pre-treatment prevents 6-OHDA toxicity in SHSY5Y cells. **A** Phase contrast microscopy of SHSY5Y cells was exposed to 50 µM of 6-OHDA for mimicking the neurodegeneration of PD. To assess the neuroprotective ability of NPs against 6-OHDA induced cytotoxicity, cells were pre-treated with 100 nM of the hormones 24 h prior to 6-OHDA addition to the cell culture medium. Bars 100 µm. **B** Cell viability assay performed on SHSY5Y cells after 19 h of 6-OHDA treatment by Trypan blue dye exclusion method; results reported as a percentage of live/dead cells, are mean ± SD from three independent experiments (*n* = 3). Significance (one-way ANOVA test + Tukey post-test): ^*^vs. untreated control (CTR), ^#^vs. 6-OHDA; ^**^p < 0.01, ^***^p < 0.001. Additional results from the one‐way ANOVA test used to analyze the differences between groups are reported in Supplementary Data. **C** Quantitative assessment of the protective effect exerted by NPs on neurite network performed on phase-contrast microscopic images, by counting the number of neurites/cell in treated and untreated SHSY5Y cells. A minimum of 300 cells/sample has been analyzed and results were reported as mean values ± SD from three independent experiments. Significance (two-tailed Student’s *t* test) ^*^vs. untreated control (CTR), ^**#**^vs. 6-OHDA; ^*^p < 0.05, ^**^p < 0.01.

To assess the ability of BNP and CNP in preventing neurotoxin-induced toxicity in SHSY5Y cells, as previously demonstrated for ANP [55], cells were pre-incubated with the NPs 24 h before the addition of 50 µM 6-hydroxydopamine (6-OHDA) to cell cultures and analyzed after additional 19 h for morphological changes and cell viability. The effects were compared with those of ANP and with the untreated control (Fig. 2). 6-OHDA treatment consistently decreased the number of adhering cells and destroyed the neurite network in SHSY5Y cells (Fig. 2A), also significantly increasing the percentage of dead cells compared with the untreated control (Fig. 2B). The exposure to NPs alone did not influence cell adhesion and viability nor neurite integrity (not shown). Pre-treatments with BNP and CNP, similarly to ANP, were able to prevent cell detachment from the substrate, also substantially preserving the neuritic network (Fig. 2A), and significantly reduced the percentage of dead cells induced by the 6-OHDA challenging (Fig. 2B).

### NPs activate canonical Wnt signaling β-catenin and prevent neurotoxin-induced toxicity in primary cultures of DA neurons

**Fig. 3:**
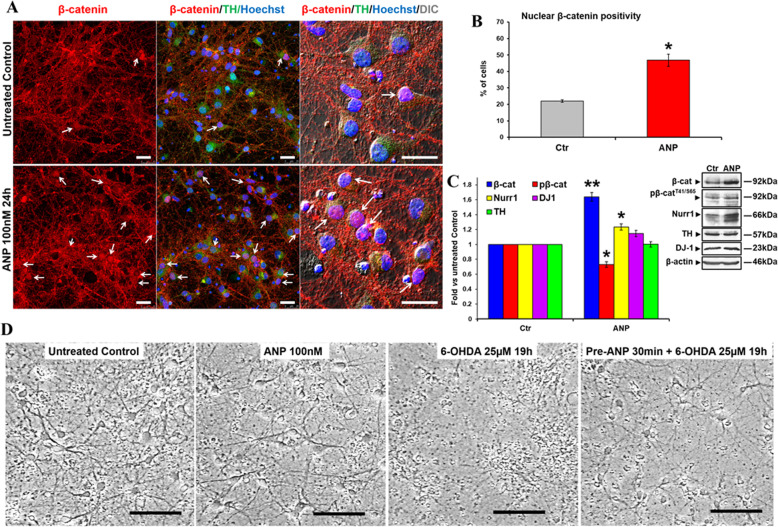
ANP induces β-catenin stabilization and nuclear translocation and is neuroprotective in primary cultures of DA neurons from mouse brain. **A** Confocal microscopy images showing the intracellular distribution of β-catenin (*red hue*) in control and ANP treated DA neurons (TH positive; *green hu*e) after 24 h; double immunofluorescent staining β-catenin/TH. Nuclei were visualized by Hoechst counterstaining (*blue hue*). Merged images with differential interference contrast (DIC), used for visualizing cell morphology, are also shown. Arrows point to β-catenin positive nuclei in TH positive cells. Bars 25 µm. **B** Quantitative evaluation of the percentage of TH positive cells exhibiting nuclear β-catenin, in untreated and ANP treated cultures. Data were obtained by counting a minimum of 300 cells/samples and results showed are the mean ± SD from three independent experiments (*n* = 3). Significance vs. untreated control (two-tailed Student’s *t* test): ^*^p < 0.05. **C** WB and densitometric analysis of total and phosphorylated β-catenin (pβ-catenin^T41/S65^), TH, Nurr-1, and DJ-1 levels in total cell lysates from ANP treated cells, compared to the untreated control, after 24 h of culture. In the densitometric analysis, values were normalized to β-actin, or vs. the total levels of β-catenin for the phosphorylated form. Results are the mean ± SD from three independent experiments (*n* = 3). Significance vs. control (two-tailed Student’s *t* test): ^*^p < 0.05; ^**^p < 0.01. **D** Phase contrast microscopy of primary DA neurons exposed to 25 µM of 6-OHDA for mimicking the neurodegeneration of PD. To assess the neuroprotective ability of NPs against 6-OHDA induced cytotoxicity, cells were pre-treated with 100 nM of the hormone 30 min prior to 6-OHDA addition to the cell culture medium. Bars 100 µm.

**Fig. 4:**
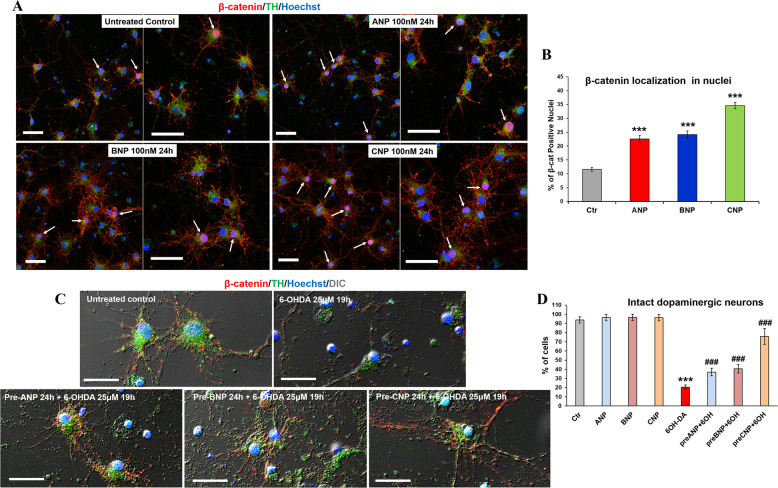
BNP and CNP induce β-catenin nuclear translocation and exhibited protective ability against 6-OHDA induced toxicity in primary cultures of DA neurons. **A** Confocal microscopy images showing the intracellular distribution of β-catenin (*red hue*) in control and NPs treated DA neurons (TH positive; *green hue*) after 24 h of culture. Merged images of β-catenin/TH double immunofluorescent staining and nuclei counterstaining with Hoechst (*blue hue*) are shown. Arrows point to β-catenin positive nuclei in TH-positive cells. Bars 50 µm. **B** Quantitative evaluation of the percentage of TH-positive cells exhibiting nuclear β-catenin, in untreated and NPs treated cultures. Data were obtained by counting a minimum of 300 cells/samples and results showed are the mean ± SD from three independent experiments (*n* = 3). Significance vs. untreated control (one-way ANOVA test + Tukey post-test): ^***^p < 0.001. **C** Confocal microscopy of primary DA neurons exposed to 25 µM of 6-OHDA for mimicking the neurodegeneration of PD. To assess the neuroprotective ability of NPs against 6-OHDA induced cytotoxicity, cells were pre-treated with 100 nM of the hormones 24 h prior to the neurotoxin challenge. Double immunofluorescent staining of β-catenin (*red hue*) and TH (*green hue*) was performed. Nuclei were visualized by Hoechst counterstaining (*blue hue*). Merged images with differential interference contrast (DIC), used for visualizing cell morphology, are shown. Bars: 25 µm. **D** Quantitative evaluation of the percentage of intact dopaminergic cells in primary cultures subjected to neurotoxin insult in presence of NPs and in those challenged in NPs-free medium. Data from untreated control or cells treated with NPs in absence of neurotoxin insult are also reported. A minimum of 300 nuclei/samples were counted and the results showed are the mean ± SD from three independent experiments (*n* = 3). Significance (one-way ANOVA + Tukey multiple comparison tests): ^*^vs. untreated control (CTR), ^#^vs. 6-OHDA; ^***^p < 0.001; the mean ± SD; *n* = 3. Additional results from the one‐way ANOVA test used to analyze the differences between groups are reported in Supplementary Data.

We have previously demonstrated that ANP has the neuroprotective ability by activating the Wnt/β-catenin pathway by using in vitro models of PD performed on SHSY5Y cells both in basal condition or differentiated by RA [55]. In order to extend this ANP property to mature DA neurons, we assessed, in the first set of experiments, the capacity of this peptide in affecting the canonical Wnt signaling and in exerting neuroprotection against 6-OHDA challenging by using, as a cellular model, primary cultures of DA neurons obtained from mouse brain, as previously described [57]. These cells homogeneously exhibited neuronal shape and β-catenin mainly distributed at the cell membrane, and all living and intact cells were positive for the dopaminergic marker TH (Supplementary Fig. S1). In these primary DA neurons, the treatment with ANP induced nuclear translocation of β-catenin (Fig. 3A), with a significant increase in the percentage of cells exhibiting β-catenin positive nuclei compared to the untreated control (Fig. 3B). WB analysis (Fig. 3C) showed that similar to what was previously observed in SHSY5Y cells, ANP induces an increase of total β-catenin, and a concomitant decrease in β-catenin phosphorylation at Thr41/Ser65 (pβ-catenin^T41/S65^), indicative of β-catenin stabilization. Concomitantly, a lower but significant increase in the expression levels of the DA neuron-specific marker Nurr1 was recorded, even if DJ-1 and TH were not significantly modified (Fig. 3C). Phase-contrast microscopic observation, performed on primary cultures subjected to 6-OHDA insult, showed that 30 min pre-treatment with ANP prevented neurotoxin-induced cell detachment and preserved the neuritic network, thus confirming the neuroprotective ability of this hormone also in this fully differentiated cellular system (Fig. 3D). Moreover, results from the second set of experiments showed that, in these primary cultures of DA neurons, also BNP and CNP, similarly to ANP used as a positive control, were able to induce β-catenin nuclear translocation (Fig. 4A, B), and exhibited protective ability against 6-OHDA induced toxicity (Fig. 4C, D). This is demonstrated by the significant increase in the number of TH^+^ cells exhibiting β-catenin in the nuclei in NPs treated cells compared with the untreated control (Fig. 4A, B), and by the higher number of residual TH^+^ cells in the cultures subjected to neurotoxin insult in presence of NPs compared with those challenged in NPs-free medium (Fig. 4C, D).

### NPs improve DA neuronal differentiation and maturation of partially committed hiPSCs

**Fig. 5:**
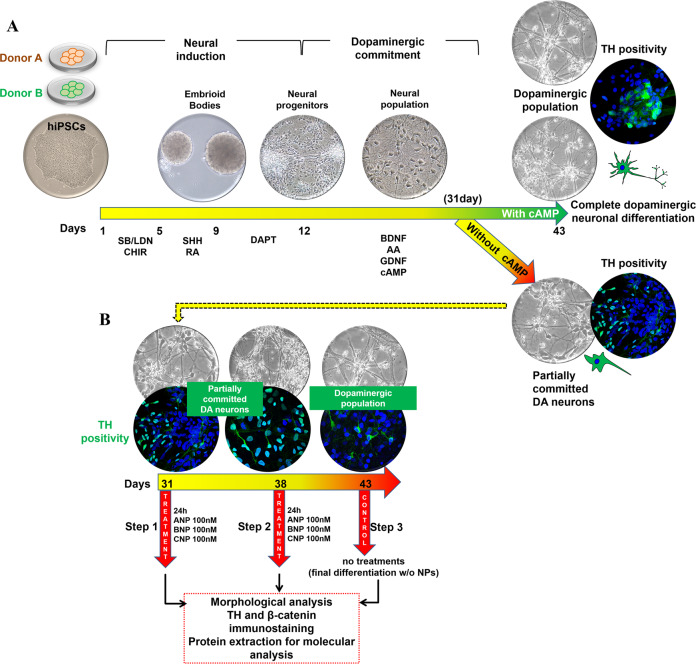
Representative scheme illustrating the generation of committed DA neuron from hiPSCs, the DA neuronal differentiation by NPs treatments, and the analyses performed. **A** hiPSCs obtained from two different healthy donors (Donor A and Donor B) were used. Neural induction (day 1–12) and dopaminergic commitment (day 12–30) have been obtained as previously reported [59], and described in the Materials and Methods section. Complete dopaminergic neuronal differentiation was reached at day 43 of culture in presence of cAMP. **B** At day 30, partially committed DA populations from both donors A and B were maintained in cAMP-free culture medium and treated, at day 31 (Step 1, analyzed at day 32) or at day 38 (Step 2, analyzed at day 39), for 24 h with 100 nM of NPs. The NPs treatments have been performed at day 31 or at day 38 for assessing if the effects produced could be dependent on the state of intrinsic differentiation of the committed neurons. Cultures maintained in cAMP- and NPs-free culture medium from day 30 to day 43 (Step 3) was used as control of cells grown in absence of factors inducing DA neuronal differentiation.

**Fig. 6:**
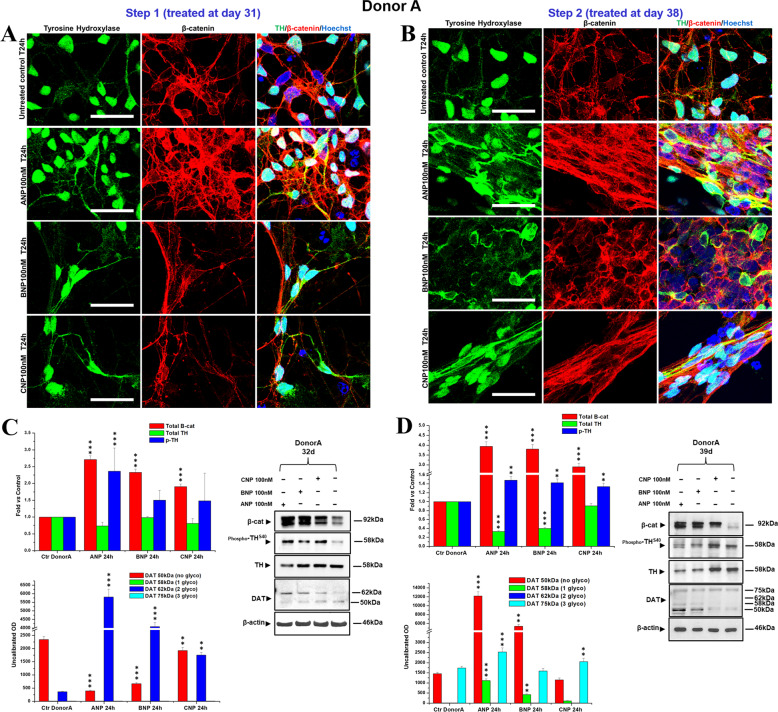
NPs induce dopaminergic differentiation in a hiPSCs-derived neuronal population obtained from DONOR A. **A**, **B** Confocal microscopy images showing the effect of 24 h treatments with NPs, performed at day 31 (Step 1; **A**) or at day 38 (Step 2; **B**) of differentiation, on the expression and intracellular distribution of TH (*green hue*) and β-catenin (*red hue*), compared with the untreated control. Merged images of β-catenin/TH double immunofluorescent staining and nuclei counterstaining with Hoechst (*blue hue*) are also shown. Bars 25 µm. **C**, **D** WB analyses of the effects of NPs treatments, at day 31 (Step 1; **C**) or at day 38 (Step 2; **D**) of total β-catenin, total and phosphorylated TH (pTH at Ser40), and of unglycosylated (50 kDa), mono- (58 kDa), di- (62 kDa), and tri- (75 kDa) glycosylated forms of the dopamine transporter DAT. In the densitometric analysis, values were normalized to β-actin, or vs. the total levels of total TH for phosphoTH^S40^. Results are the mean ± SD from three independent experiments (*n* = 3). Significance vs. the untreated control (one-way ANOVA + Tukey multiple comparison test): ^**^p < 0.01, ^***^p < 0.001. Additional results from the one‐way ANOVA test used to analyze the differences between groups are reported in Supplementary Data.

**Fig. 7:**
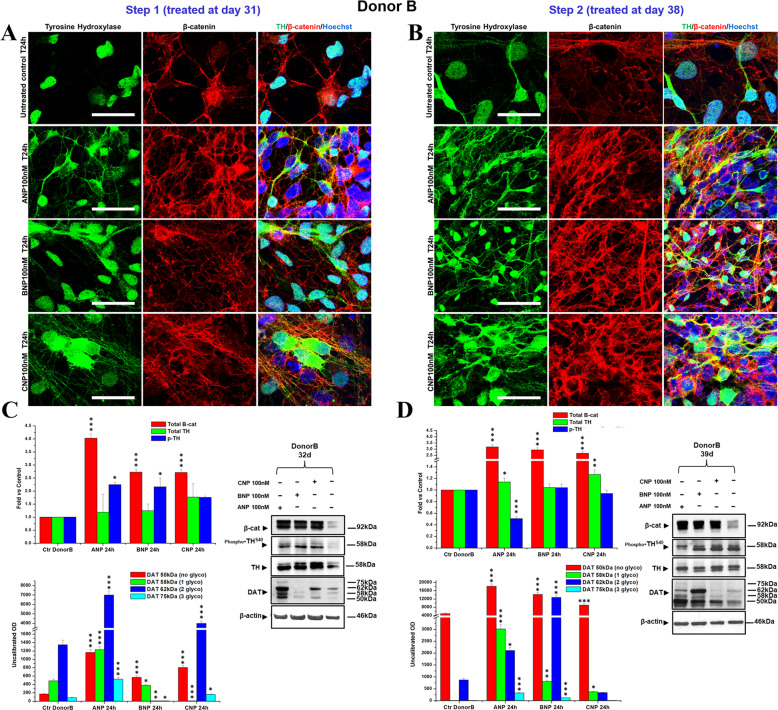
NPs induce dopaminergic differentiation in a hiPSCs-derived neuronal population obtained from DONOR B. **A**, **B** Confocal microscopy images showing the effect of 24 h treatments with NPs, performed at day 31 (Step 1; **A**) or at day 38 (Step 2; **B**) of differentiation, on the expression and intracellular distribution of TH (*green hue*) and β-catenin (*red hue*), compared with the untreated control. Merged images of β-catenin/TH double immunofluorescent staining and nuclei counterstaining with Hoechst (*blue hue*) are also shown. Bars 25 µm. **C**, **D** WB analyses of the effects of NPs treatments, at day 31 (Step 1; **C**) or at day 38 (Step 2; **D**) of total β-catenin, total and phosphorylated TH (pTH at Ser40) and of unglycosylated (50 kDa), mono- (58 kDa), di- (62 kDa), and tri- (75 kDa) glycosylated forms of the dopamine transporter DAT. In the densitometric analysis, values were normalized to β-actin, or vs. the total levels of total TH for phosphoTH^S40^. Results are the mean ± SD from three independent experiments (*n* = 3). Significance vs. the untreated control (one-way ANOVA + Tukey multiple comparison test): ^*^p < 0.05, ^**^p < 0.01, ^***^p < 0.001. Additional results from the one‐way ANOVA test used to analyze the differences between groups are reported in Supplementary Data.

**Fig. 8:**
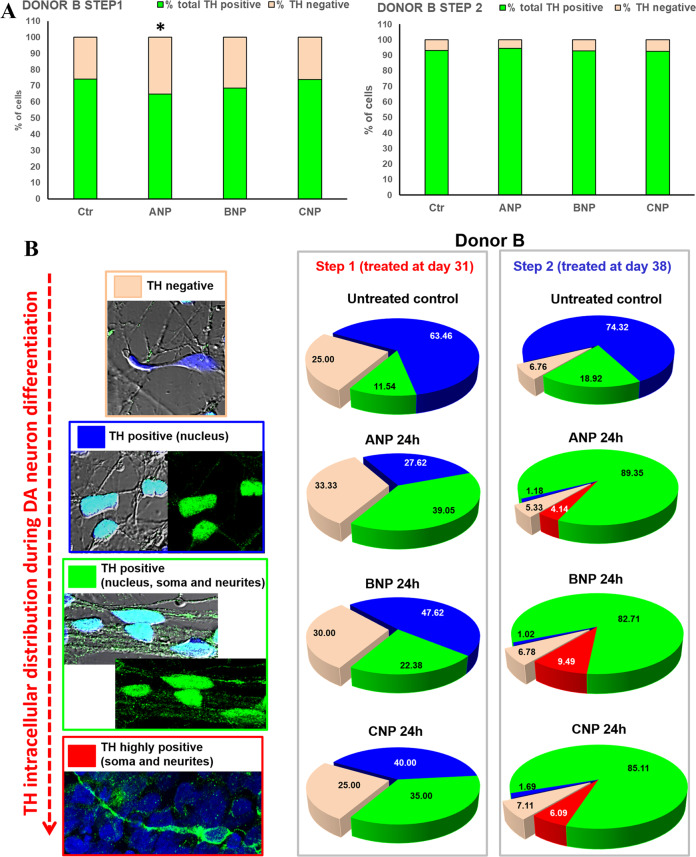
NPs increase the proportion of DA neurons expressing TH in the soma and along the axons and neurites: hiPSCs-derived neuronal population obtained from DONOR B. **A** Quantitative evaluation of the percentage of TH^+^ cells in cultures treated at day 31 (Step 1) or at day 38 (Step 2) with NPs, compared with the untreated controls. Data were obtained by counting a minimum of 300 cells/samples and results showed are the mean ± SD from three independent experiments (*n* = 3). Significance vs. untreated control (two-tailed Student’s *t* test): ^*^p < 0.05. **B** *Left panels*: representative images by confocal microscopy showing the modification of the intracellular distribution of total TH (*green hue*) observed during NPs-induced dopaminergic differentiation. *Right panels*: Quantitative evaluation of NPs effect on the percentage of TH^−^ cells and of TH^+^ neurons exhibiting this enzyme exclusively in the nucleus (poorly differentiated), in the nucleus, soma, and neurites (early differentiated) or exclusively in soma and neurites (mature DA neurons).

HiPSC lines previously generated [58] from two different healthy donors (Donor A and Donor B) exhibited an undifferentiated morphology with compact refractile, defined borders, and a high rate of proliferation [58] (Fig. 5A). hiPSCs from both donors have been differentiated into DA neurons [59] as described in the Materials and Methods, resumed in Supplementary Table 1 and schematized in Fig. 5A. In our experimental conditions, complete dopaminergic neuronal differentiation was reached at day 43 of culture in presence of cAMP. At day 30, cultures had lost the neural stem cells population, as confirmed by the negativity for the early marker of neural stem cell Nestin, and had acquired phenotypic features of the partially committed dopaminergic population, as demonstrated by the positivity for the neural and dopaminergic markers tubulin β3, TH, and Nurr1 (Supplementary Fig. S2). For assessing the ability of NPs in stimulating differentiation and maturation toward the dopaminergic linage, at day 30 cAMP was removed from the culture medium, and the two batches of the neural population (one from Donor A and one from Donor B) were treated in parallel, at day 31 (Step1) or at day 38 (Step2), for 24 h with 100 nM ANP, BNP, or CNP, as described in Materials and Methods and schematized in Fig. 5B. Cultures maintained in cAMP- and NPs-free medium from day 30 to day 43 (Step 3) were used as untreated controls. A neural population cultured until day 43 in presence of cAMP was used as control of DA neuron maturation. The effects of NPs indicative of β-catenin signaling activation have been assessed by analyzing β-catenin intracellular distribution and expression. NPs-induced improvement of dopaminergic differentiation has been verified by analyzing cell morphology, the number of TH^+^ cells, total TH intracellular distribution and expression as well as the levels of TH phosphorylation at Ser40, that positively regulates the catalytic activity of this enzyme involved in dopamine biosynthesis. To further assess if NPs were able to improve DA neurons functionality and maturation, the expression and glycosylation levels of the dopamine transporter (DAT), the transmembrane protein responsible of dopamine reuptake at synaptic terminations, considered the gold standard marker for the identification of mature DA neurons, were also analyzed. After 24 h of treatment with ANP, BNP, or CNP, partially committed DA neurons exhibited morphological features suggestive of complete dopaminergic neuronal differentiation already at day 32 (Step 1, treated at day 31) or at day 39 (Step 2, treated at day 38), similar to what observed later, at day 43, in cultures maintained in NPs-free medium but in presence of cAMP (Supplementary Fig. S3). Under NPs treatments performed both at Step 1 or at Step 2, a dramatic and highly significant increase in β-catenin expression, with a concomitant accumulation in the cytoplasmic and nuclear compartments, was also observed in the cultures from both donors A and B, even if with some quantitative but not qualitative differences between the two donors (Figs. 6 and 7, Supplementary Figs. S4–S6). Moreover, in both donors and at both steps of treatment, all NPs significantly increased the proportion of TH^+^ neurons expressing this enzyme both in the soma and along the axons and neurites (Figs. 6A, B and 7A, B, Supplementary Figs. S4 and S5), features that have been reported to distinguish the mature DA neurons from the other catecholaminergic TH^+^ populations [60]. This was confirmed by the quantitative analysis of the subcellular localization of TH in NPs treated cells vs. the untreated controls. Indeed, even if a significant increment in the total number of TH^+^ cells was only recorded in the Donor A (Fig. 8A and Supplementary Fig. S5A), in both donors and at both steps analyzed, the percentage of cells expressing TH in soma and neurites increased under NPs treatments, while in the untreated controls the majority of cells exhibited also or exclusively nuclear TH positivity (Fig. 8B and Supplementary Fig. S7B). Since it has been reported that nuclear localization of TH is related to its proteasomal degradation, triggered by phosphorylation at Ser19, while the active enzyme has cytoplasmic localization [61, 62], our observations suggest that the different TH localization observed in controls and in NPs treated cultures could be related to catalytically inactive (nuclear) or catalytically active (soma and neurites) enzyme, the latter increasing during NPs-induced differentiation. Indeed, in cAMP-treated positive control of dopaminergic neurons at day 43, TH mainly, but not exclusively, localized in the cytoplasm (Supplementary Fig. S8). This was further confirmed by the concomitant increase of TH phosphorylation levels at Ser40 in NPs treated cultures from both donors compared with the untreated controls, indicating that the three hormones were able to improve the catalytic activity of this enzyme, positively affecting the dopamine biosynthesis (Figs. 6C, D and 7C, D). The NPs-stimulated improvement of mature DA neuron phenotype was further reinforced by the higher expression of DAT and its glycosylated forms recorded in treated vs. the untreated cultures obtained from both donors, even if with some quantitative but not qualitative differences (Figs. 6C, D and 7C, D). It has been reported that DAT glycosylation progressively increases throughout development [63], stabilizes DAT localization at the plasma membrane, and increases the dopamine transport rate, while the non-glycosylated form inefficiently transports dopamine compared with the fully glycosylated ones [64]. Thus, the increase in the levels of DAT glycosylated forms vs. the non-glycosylated one recorded in treated vs. the untreated cultures from both donors, confirms that the treatments with all the three NPs, even if more markedly for ANP and BNP than for CNP (Figs. 6C, D and 7C, D), stimulate differentiation of partially committed neuronal cells toward mature and functional DA neurons.

## Discussion

Over the last twenty years, the efforts to develop new therapies for PD have focused not only on the improvement of symptomatic therapy for motor and non-motor symptoms but also on the discovering of the potential causes of PD, in order to develop a disease-modifying treatment. On this ground, the emerging involvement of the Wnt/β-catenin pathway dysregulation in the onset and progression of PD renders the targeting of this signaling an attractive therapeutic opportunity for curing this brain disorder. The demonstrated role of the Wnt/β-catenin signaling in adult neurogenesis [21, 25, 26] reinforces the possibility that molecules targeting this signaling could be effective not only in arresting the progression of PD but also to pharmacologically stimulating the endogenous neural stem cell niches and counteract the loss of the neurons [65]. Thus, targeting the canonical Wnt signaling appears as a potentially innovative strategy not only for neuroprotection but also for neurorepair and cell replacement in PD [27, 28, 35, 38, 66–68].

Here, we demonstrate that the three NPs, ANP, BNP, and CNP, possibly by functioning as Wnt/β-catenin signaling modulators, could be promising molecules for developing a PD-modifying therapy. Specifically, we first confirm that BNP and CNP, as previously demonstrated for ANP [55], are able to positively affect the canonical Wnt signaling and to be neuroprotective for SHSY5Y cells, used as a model of DA neurons, even if CNP seems to be active at later times (Figs. 1 and 2). Further, all the three NPs stabilize β-catenin and are neuroprotective on primary cultures of DA neurons from the mouse brain, and this demonstrates their efficacy also in a fully differentiated cellular system (Figs. 3 and 4). Finally, and more importantly, ANP, BNP, CNP, are able to improve and accelerate the dopaminergic differentiation and maturation of partially committed neurons from hiPSCs and this process is closely correlated to β-catenin stabilization, cytosolic accumulation, and nuclear translocation (Figs. 6 and 7). These effects have been recorded in hiPSCs-derived neural populations obtained from two differed healthy donors, that exhibited a similar pattern of differentiative response under NPs stimulation, even if with some quantitative but not qualitative differences. Specifically, in the neural population from Donor A the phosphorylation levels of TH at Ser40 as well as the expression and glycosylated forms of DAT, are increased under treatments with all the three NPs and at both Step 1 and Step 2 (Fig. 6C, D). Instead, in the neural population from Donor B, posphoTH^Ser40^ increased when we treated cells after 31 days of culture (Step 1) but not when we treated them one week later (day 38, Step2), while DAT glycosylation levels at both Step 1 and Step 2, with the exception of BNP treatment, for which a dramatic increase in DAT expression/glycosylation was recorded only when we treated cells at day 38 of culture (Fig. 7C, D). The few differences recorded in the NPs-induced effects on the two neuronal populations could be possibly related to the intrinsic variability of the hiPSCs lines from each donor, which might affect the final yield of DA neurons (Supplementary Fig. S9). Indeed, at the time when we performed the NPs treatments (31 days), the neural population from Donor B appeared morphologically and phenotypically more differentiated than the neural population from Donor A, as suggested by the higher levels of total TH and of the glycosylated forms of DAT in the untreated controls form Donor B vs. controls from Donor A (Figs. 6C and 7C), and this could affect the quantity and the timing at which the effects were recorded. In any case, in both cell populations we recorded, on the overall, an increase of phosphorylation levels of TH at Ser40 and an increase of DAT expression and/or glycosylation levels, and these effects, taken together, strongly suggest that the NPs stimulate the differentiation of neuronal cells from both donors towards mature and functional DA neurons. However, additional studies are needed to confirm the NP’s ability in promoting neural differentiation and maturation towards the dopaminergic linage also in hiPSCs-derived lines obtained from PD patients. The therapeutic significance of endogenous neurogenesis for the recovery of old-onset PD or NDs remains to be elucidated but it is currently actively investigated. Numerous recent works support the upregulation of the Wnt/β-catenin signaling as a means to reactivate neurogenesis and stimulate the inherent self-repair capacities in the injured brain, and both pharmacological and cellular therapies based on activation of this signaling were reported to promote endogenous neurogenesis and to improve neuro-restoration in the aged PD brain [65]. On this ground, our results reinforce the attractiveness and the therapeutic potential of NPs.

Even if the mechanism through which NPs trigger the Wnt/ß-catenin signaling is not been yet investigated and will be deepened in future studies, for BNP we suggest that it could mainly involve an Fzd-related modality, as previously demonstrated for ANP [53], rather than acting through the specific receptor NPR-A. Indeed, the Fzd1 and Fzd2 structural domains enclosed in the extracellular region of Corin are crucial for both pro-ANP and pro-BNP processing [44], and this makes reasonable a direct interaction of ANP and BNP with the Wnt signaling receptors. Instead, since the processing of pro-CNP does not involve Corin, the CNP-induced β-catenin stabilization and nuclear translocation could be secondarily triggered by the cyclic GMP response elicited through the interaction with its specific receptor NPR-B. Even if further studies will be necessary to specifically clarify this point, the differences in the timing and intensity of the effects induced by ANP and BNP (quantitative and qualitative very similar) from those recorded under CNP treatment, are suggestive of different mechanisms through which the Wnt/ß-catenin signaling is triggered.

Apart from the possible therapeutic implication, modulation of the Wnt signaling could also be one of the functions of endogenous NPs in the brain. It is known that the balancing between *Wnt-on* and *Wnt-off* in dopaminergic neurons is controlled by the microglial/astrocytic component of the midbrain [27, 28, 69, 70], and astroglial and microglial cells have been reported to produce and release ANP, BNP, and CNP, and to express functional NPRs [49]. Thus, microglial/astrocytic systems seem to be both potential sources and targets of NPs that in turn exert autocrine or paracrine actions on glial cells and on surrounding neurons. Furthermore, the NP–NPR system of glial cells has also been suggested to participate in neuron/glia communication and could have crucial roles in regulating neuroinflammation and neuroprotection [49]. Our results thus suggest that the regulatory role of NPs in the midbrain could be possibly mediated not only by the binding to the specific NPRs and activation of the cGMP-dependent signaling, as previously reported [48, 49], but also by modulation of the Wnt pathway.

In conclusion, in this work, we gained in vitro evidence supporting the relevance of exogenous ANP, BNP, and CNP as attractive protective and neuroreparative molecules for PD, and more in general, for brain diseases for which aberrant Wnt signaling seems to be the leading pathogenetic mechanism. The attractiveness of these NPs for developing a possible PD-modifying therapeutic approach is reinforced by the fact that human recombinant ANP (Carperitide), BNP (Nesiritide, BNP-32), and CNP (Vosiritide) have already entered in clinical trials for the treatment of heart diseases/dysfunctions and hypertension and, *de facto*, have already passed the toxicological screening for extension of the therapeutic application of these molecules. Even if further studies will be required to exhaustively demonstrate the effectiveness in neuroprotection and neurorepair of these NPs and to deepen the mechanism through which the canonical Wnt signaling is involved, our results establish a stimulating starting point for a possible future application of these hormones for counteracting the progression of PD and other NDs which onset has been related to aberrant canonical Wnt signaling.

## Materials and methods

### Cell cultures

SHSY5Y cell line was provided by the American Type Culture Collection (ATCC, Manassas, VA, USA) and was validated by the ATCC cell bank. Cells were grown as a monolayer in Eagle’s Minimum Essential Medium (α-MEM) plus HAM’s F12 (1:1), supplemented with 10% heat-inactivated fetal bovine serum (FBS), l-glutamine (2 mM), penicillin (100 IU/ml), and streptomycin (100 µg/ml), and maintained at 37 °C, in a humidified atmosphere of 5% CO_2_. For passaging, cells were detached from culture flasks with 0.05% trypsin and 0.002% EDTA solution. All media and supplements for cell cultures were acquired from Hyclone (Logan, UT, USA).

Primary cultures of mouse DA neurons were kindly provided by Maria Teresa Ciotti (Institute of Cellular Biology and Neurobiology, National Research Council of Italy), obtained from dissected brains of 13-day-old mouse embryos, as previously published [57]. For treatments, cells were plated at a density of 6 × 10^4^ cells/cm^2^ and maintained in a Neurobasal medium (Invitrogen) supplemented with B-27.

HiPSC lines have been generated reprogramming skin biopsy-derived fibroblasts from healthy donors (Donor A and Donor B) as previously described [58], using the polycistronic hSTEMCCA-loxP lentiviral vector expressing the four Yamanaka’s factors (hOct4, hSox2, hKlf4, and hc-Myc) [71]. Characterization of hiPSCs colonies has been performed as previously published [58], by analyzing the expression of the endogenous pluripotency marker genes (OCT4, SOX2, REX1, Klf4, c-Myc, and NANOG) by RT-PCR analysis. HiPSC lines were manually picked, passaged on human embryonic stem cell-qualified Matrigel-coated plates (0.05 mg/mL; BD Biosciences), and cultured under the feeder-free condition in mTeSR1 medium (Stem Cell Technologies) with Y-27632 ROCK inhibitor (Stemcell Technologies), that maintains the stability over 30 and more passages. The stemness propriety and karyotype analysis have also been assessed (not shown). HiPSCs have been then differentiated into DA neurons by using an embryoid body (EB) formation protocol [59, 72], on low attachment cell culture plates, as schematized in Fig. 5A. For neural induction, hiPSCs were cultured, on day 1-17, in Neurobasal medium/DMEM F-12 (1:1) supplemented with B-27 (1×), N-2 (1×), Pen/Strep (1%), NEAA (1%) and β-Mercaptoethanol (0,1%) (Supplementary Table 1). On day 2–4, this complete culture medium was added with the GSK-3 inhibitor CHIR99021 [1 μM], and combining dual ALK inhibition by (SB431542 [40 μM] (Santa Cruz Biotechnology), LDN193189 [0.2 μM] (Stemgent). On days 5–7, the medium was replaced and added with sonic hedgehog [500 nM] (SHH-Curis) and retinoic acid (RA) [10 nM]. On days 8–17, the differentiation medium was replaced, and supplemented with DAPT [10 µM], and changed every 2 days (Supplementary Table 1). Floating EBs were kept in suspension from day 1 to day 11, then dissociated with Accutase (Stemcell Technologies), and cells obtained were put in adhesion on the coating of poly-ornithine/laminin (*n* = 125 × 10^3^ cells/cm^2^). Following this protocol, full neural conversion from hiPSCs into neural progenitors was achieved on days 1–9. Neural population enrichment was then achieved by the addition to the Neurobasal medium, on days 18–43, of BDNF [20 ng], ascorbic acid [0.2 mM], GDNF [20 ng], for neuronal differentiation, plus cAMP [0.5 mM] for the commitment toward the dopaminergic subtype. (Supplementary Table 1). A total of 3000 EBs for donors (A and B) were used and the same monolayer confluence was got for each treatment.

### Treatments

Exponentially growing SHSY5Y cells were seeded at densities of 4 × 10^4^/cm^2^ and cultured for 24 h prior to treatments. Primary cultures from the murine brains were maintained in culture for 13 days to obtain mature DA neurons. SHSY5Y cells and primary DA neurons were treated with 100 nM ANP, BNP, or CNP (Phoenix Pharmaceuticals Inc.), a concentration previously selected for ANP as the lowest effective and not toxic dose in dose-response experiments [55], for times ranging from 6 to 24 h.

For mimicking the neurodegenerative features of PD, SHSY5Y cells, and primary cultures of DA neurons were exposed for 24 h to 50 µM and 25 µM of 6-OHDA (prepared in 0.1% ascorbic acid/DMSO), respectively, concentrations selected in preliminary dose-response experiments as the lowest effective doses. For verifying the neuroprotective ability of the three NPs, both cellular systems were pre-incubated with 100 nM ANP, BNP or CNP, 30 min or 24 h before adding 6-OHDA to cell cultures, and analyzed after an additional 19 h for morphology and cell viability, and for the expression levels of β-catenin, dopaminergic neuron markers, and survival factors.

To assess the capacity of NPs in stimulating the hiPSCs differentiation toward the dopaminergic linage, two EB batches, each obtained from two healthy donors (Donor A and Donor B), were placed in adhesion as described above and maturated on days 12–30 with BDNF, ascorbic acid, and GDNF, plus cAMP for the commitment towards DA neuron phenotype. For evaluating the efficacy of NPs treatments, at day 30 cAMP was removed from the culture medium, and each batch of the neural population was treated in parallel, at day 31 or at day 38, for 24 h with 100 nM ANP, BNP, or CNP. as schematized in Fig. 5B. Cells maintained until day 43 in NPs- and the cAMP-free medium were used as untreated control. A neural population cultured until day 43 in presence of cAMP was used as control of DA neuron maturation. All the experiments were carried out using two batches of neural induction (one batch from hiPSCs obtained from Donor A and one batch from hiPSCs obtained from Donor B), every single treatment and controls were performed on adhering cells plated in triplicate at the same cell density and the results were the mean of three independent experiments using the same batch from each donor.

### Evaluation of cell morphology and viability, and quantitative analyses

Morphological features of treated and untreated cells were analyzed by phase-contrast microscopy, using the Motic AE31 Trinocular inverted microscope (Motic Asia, Hong Kong). The Trypan blue dye staining was used to determine cell viability. For this assay cells were seeded at densities of 4 × 10^4^/cm^2^ and cultured for 24 h before treatments. Adherent and floating cells were collected and stained with the Trypan blue dye. Quantitative evaluation of the percentage of cells exhibiting nuclear β-catenin, TH^+^ cells, and TH intracellular distribution was done on confocal microscopic images using the ImageJ processing program [http://rsbweb.nih.gov/ij/], by counting a minimum of 300 cells/sample. The quantitative assessment of the protective effect exerted by NPs on neurite network was performed on phase-contrast microscopic images, by counting the number of neurites *per* cell in treated and untreated SHSY5Y cells. Cell viability and quantitative analyses were performed on samples in triplicate and results were reported as mean values ± SD.

### Immunocytochemistry and confocal microscopy

For confocal microscopic analyses, cells were grown on the ibiTreat µ-Slide 4-well (Ibidi GmbH, Germany). Immunocytochemical analyses were performed on cells fixed with 2% paraformaldehyde (Sigma-Aldrich). After permeabilization with 0.2% Triton X-100 (Sigma-Aldrich), immunofluorescence staining was performed using the specific primary antibodies against β-catenin and TH, detailed in Supplementary Table 2. Primary antibodies were revealed with Alexa Fluor 555-conjugated anti-mouse (Invitrogen) or Alexa Fluor 488-conjugated anti-rabbit IgG (Invitrogen). The analyses were performed using the LEICA TCS SP5 confocal microscope.

### Western blot (WB) analysis

After washing with ice-cold PBS, cells were lysed using a 100 mM Tris-HCl, 300 mM NaCl, 1 mM EDTA, 200 µM EGTA, 20% glycerol, 1% NP-40, 2 mM NaVO_4_, 20 mM NaF, and a 1% protease inhibitor cocktail (Sigma-Aldrich). Protein quantification has been performed using the Bradford reagent (Bio-Rad, Segrate, Italy). Every single treatment and controls were performed on adhering cells plated in triplicate at the same cell density, and the protein extracts from each triplicate were then pooled for the WB analyses. Totally, 15–20 µg of each cell extract was separated using 8% SDS/PAGE and then transferred to nitrocellulose membrane (Hybond, Amersham GE Healthcare). Membranes were blocked 1 h at room temperature with 5% bovine serum albumin in Tris-buffered saline-Tween (TBS-T; 0.2 M Tris, 1.37 M NaCl, pH 7.6, and 0,05% Tween-20) and probed with the specific antibodies against β-catenin, pβ-catenin^Ser33/37/Thr41^, GSK-3αβ, pGSK -3β^Ser9^, TH, pTH^Ser40^, DJ-1, Nurr-1, DAT, and β-actin, detailed in Supplementary Table 2. Primary antibodies were revealed with peroxidase-conjugated secondary antibody (BioRad, Richmond, CA, USA). Densitometric analysis was performed by using the ImageJ processing program [http://rsbweb.nih.gov/ij/]. Values, normalized to β-actin or *vs* the total levels of each protein for the phosphorylated forms, were reported as fold vs. the untreated control. Data were presented as the mean from at least three independent experiments ± S.D.

### Statistical analysis

Statistical analysis was performed using the two-tailed Student’s *t* test or one‐way analysis of variance (ANOVA) followed by Tukey post hoc test, to analyze the differences between groups. A *p* value < 0.05 was assumed as statistically significant. All data were from at least three independent experiments and presented as means ± SD.

## Supplementary information

Supplementary Table 1

Supplementary Table 2

Supplementary Figure

Supplementary Data

Graphical Abstract

## Data Availability

All the data generated in this study were provided in this article, both as main and supplementary results, and the primary data files are available from the corresponding author upon reasonable request.
