# Supplementary material for: Natriuretic peptides are neuroprotective on in vitro models of PD and promote dopaminergic differentiation of hiPSCs-derived neurons via the Wnt/β-catenin signaling
Source: Cell Death Discov. 2021 Nov 1;7:330. doi: 10.1038/s41420-021-00723-6 (PMC8560781; doi:10.1038/s41420-021-00723-6)
Supplement: Supplementary file 1 — Supplementary Table 1 [file 41420_2021_723_MOESM1_ESM.docx]

**Supplementary Table 1**. Table summarizing the protocol used during neural induction of hiPSCs.

|  | **Medium** | **Supplements** |
| --- | --- | --- |
| **Day 1** | NEUROBASAL MEDIUM + DMEM/HAM’s F12 (1:1) | B-27 (1X)  N-2 (1X)  Pen/Strep (1%)  NEAA (1%)  β-mercaptoethanol (0,1%) |
| **Day 2-4** | NEUROBASAL MEDIUM + DMEM/HAM’s F12 (1:1) | B-27 (1X)  N-2 (1X)  Pen/Strep (1%)  NEAA (1%)  β-mercaptoethanol (0,1%)  ***Plus*:**  SB431542 [40 μM]  LDN193189 [0.2 µM]  CHIR99021 [1 µM] |
| **Day 5-7** |  | B-27 (1X)  N-2 (1X)  Pen/Strep (1%)  NEAA (1%)  β-mercaptoethanol (0,1%)  ***Plus*:**  Acido Retinoico [10 nM]  SHH [500 nM] |
| **Day 8-17** | NEUROBASAL MEDIUM + DMEM/HAM’s F12 (1:1) | B-27 (1X)  N-2 (1X)  Pen/Strep (1%)  NEAA (1%)  β-mercaptoethanol (0,1%)  ***Plus*:**  DAPT [10 µM] |
| **Day 18-43** | NEUROBASAL MEDIUM | B-27 (1X)  L-GLU (1X)  Pen/Strep (1%)  NEAA (1%)  ***Plus*:**  BDNF (20ng/ml)  ACIDO ASCORBICO [0.2mM]  GDNF [20ng/ml]  dibutyryl cAMP [0.5mM] |
