# Supplementary material for: Natriuretic peptides are neuroprotective on in vitro models of PD and promote dopaminergic differentiation of hiPSCs-derived neurons via the Wnt/β-catenin signaling
Source: Cell Death Discov. 2021 Nov 1;7:330. doi: 10.1038/s41420-021-00723-6 (PMC8560781; doi:10.1038/s41420-021-00723-6)
Supplement: Supplementary file 2 — Supplementary Table 2 [file 41420_2021_723_MOESM2_ESM.docx]

**Supplementary Table 2.** List of antibodies used for immunocytochemical and Western Blot analyses

| **Antigen** | **Host** | **Cat. #** | **Method/s** | **Working dilution** | **Supplier** |
| --- | --- | --- | --- | --- | --- |
| β-catenin | Mouse  (monoclonal) | 610154  (Clone 14) | IF  WB | 1:250  1:3000 | BD Transduction Labs (Palo Alto, CA, USA) |
| pβ-catenin^Ser33/37/Thr41^  (preliminary to degradation) | Rabbit (polyclonal) | 9561 | WB | 1:1000 | Cell signaling (Boston, MA, USA) |
| GSK-3αβ | Rabbit  (monoclonal) | ab16667 | WB | 1:1000 | Cell signaling (Boston, MA, USA) |
| pGSK -3β^Ser9^  (inactive form) | Rabbit (monoclonal) | 9323 | WB | 1:1000 | Cell signaling (Boston, MA, USA) |
| Tyrosine hydroxylase (TH) | Rabbit (polyclonal) | 2792 | IF  WB | 1:100  1:1000 | Cell signaling (Boston, MA, USA) |
| pTH^Ser40^ | Rabbit (polyclonal) | 2791 | WB | 1:1000 | Cell signaling (Boston, MA, USA) |
| DJ-1 | Rabbit (polyclonal) | AB9212 | WB | 1:5000 | Millipore |
| Nurr1 | Mouse (monoclonal) | Sc-81345 | WB | 1:200 | Santa Cruz Biotechnology, Inc |
| Dopamine Transporter (DAT) | Rabbit (polyclonal) | D6944 | WB | 1:2000 | Sigma-Aldrich (St. Louis, MO, USA) |
| β-actin | Mouse  (monoclonal) | A5441  (Clone AC-15) | WB | 1:10 000 | Sigma-Aldrich (St. Louis, MO, USA) |

Abbreviation used: IF: immunofluorescence, WB: Western Blot.
