# Supplementary material for: Natriuretic peptides are neuroprotective on in vitro models of PD and promote dopaminergic differentiation of hiPSCs-derived neurons via the Wnt/β-catenin signaling
Source: Cell Death Discov. 2021 Nov 1;7:330. doi: 10.1038/s41420-021-00723-6 (PMC8560781; doi:10.1038/s41420-021-00723-6)
Supplement: Supplementary file 3 — Supplementary Figure [file 41420_2021_723_MOESM3_ESM.pdf]

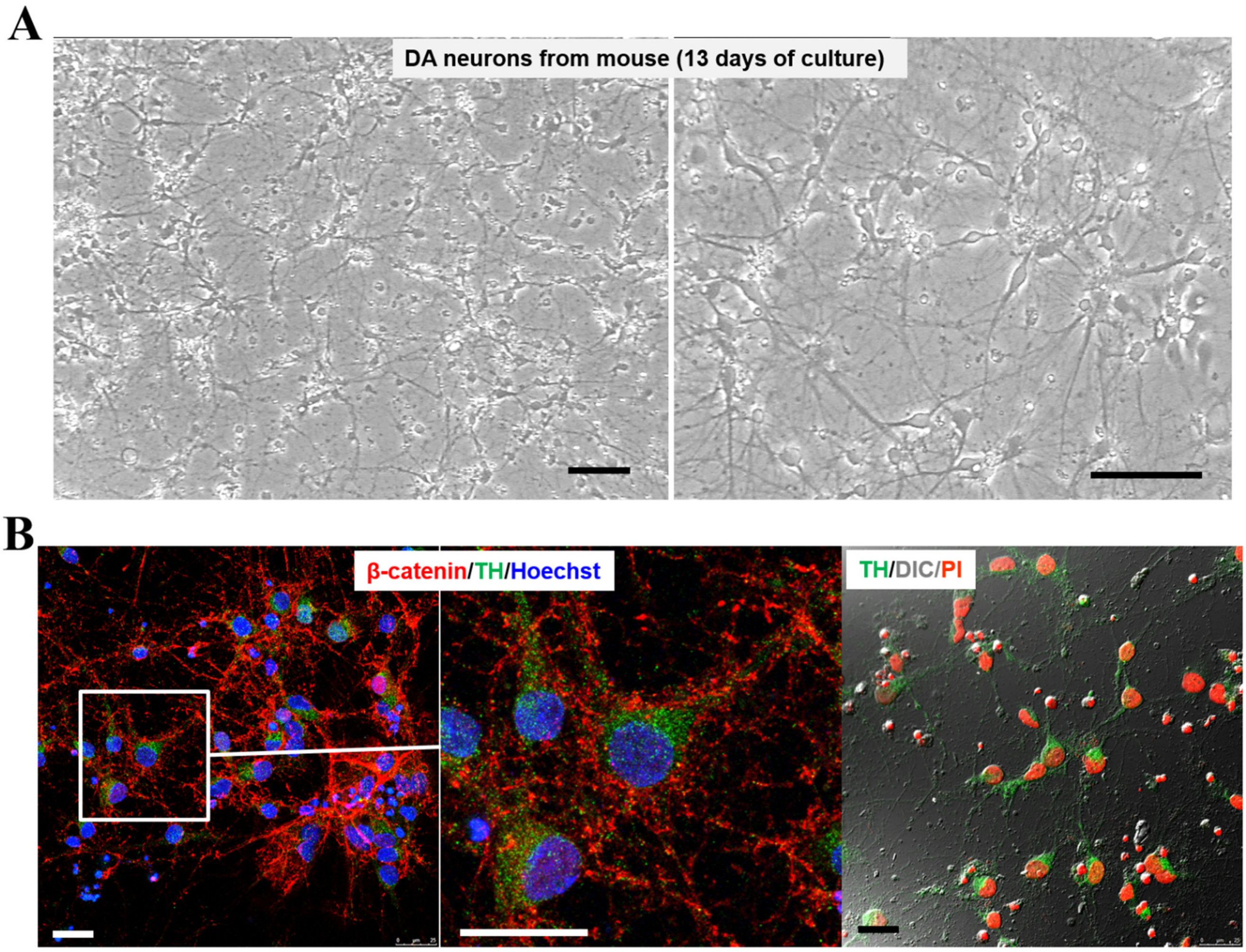

Supplementary Fig. S1. Characterization of primary cultures of DA neurons from mouse brain. A Morphology by phase contrast microscopy of primary cultures homogeneously exhibiting neuronal shape. Bars 100 µm. **B** *Left panels*: Confocal microscopy images showing the expression of the DA neuronal marker TH (*green hue*) and the intracellular distribution of β-catenin (*red hue*); cell nuclei are visualized by Hoechst counterstaining (*blue hue*). *Right panel*: Merged images of TH positive neurons (*green hue*) with differential interferential contrast (DIC), used for visualizing cell morphology; cell nuclei are visualized by Propidium Iodide (PI) counterstaining (*red hue*). Bars 25 µm.

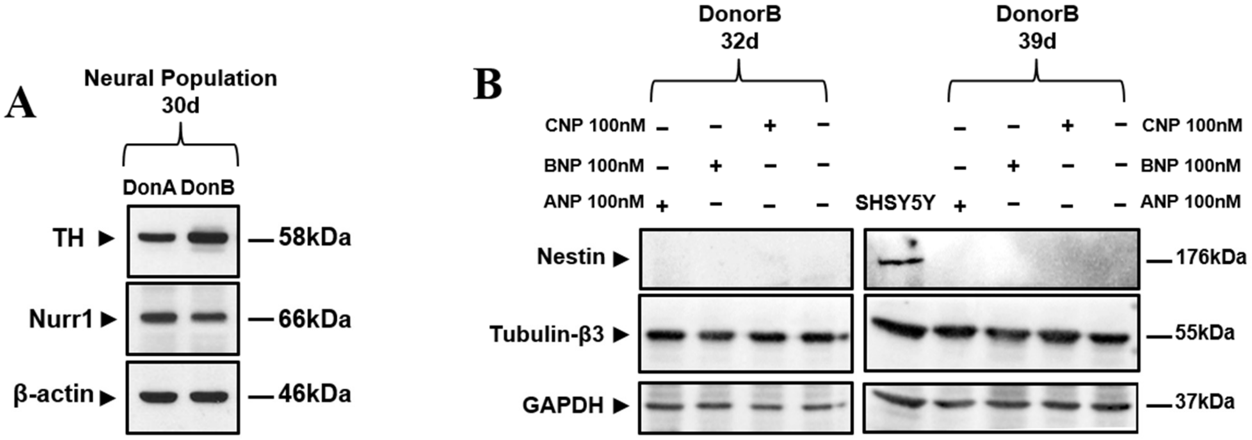

Supplementary Fig. S2. **Expression of the neural stem cell marker Nestin and of neuronal and dopaminergic markers tubulin β3, TH and Nurr1 in the hiPSCs-derived neural population. A)** WB analysis of the expression of the DA neuron markers TH and Nurr1 in neural population from Donor A and Donor B at day 30, when cultures had acquired the phenotypic features of partially committed dopaminergic population. β-actin was used as loading control. **B)** WB analysis of Nestin and tubulin β3 expression in neural population from Donor A in absence and in presence on 24h NPs treatments performed at day 31 (analyzed at day 32, Step 1) or at day 38 (analyzed at day 39, Step 2). SHSY5Y cells were used as positive control of Nestin expression. GAPDH was used as loading control.

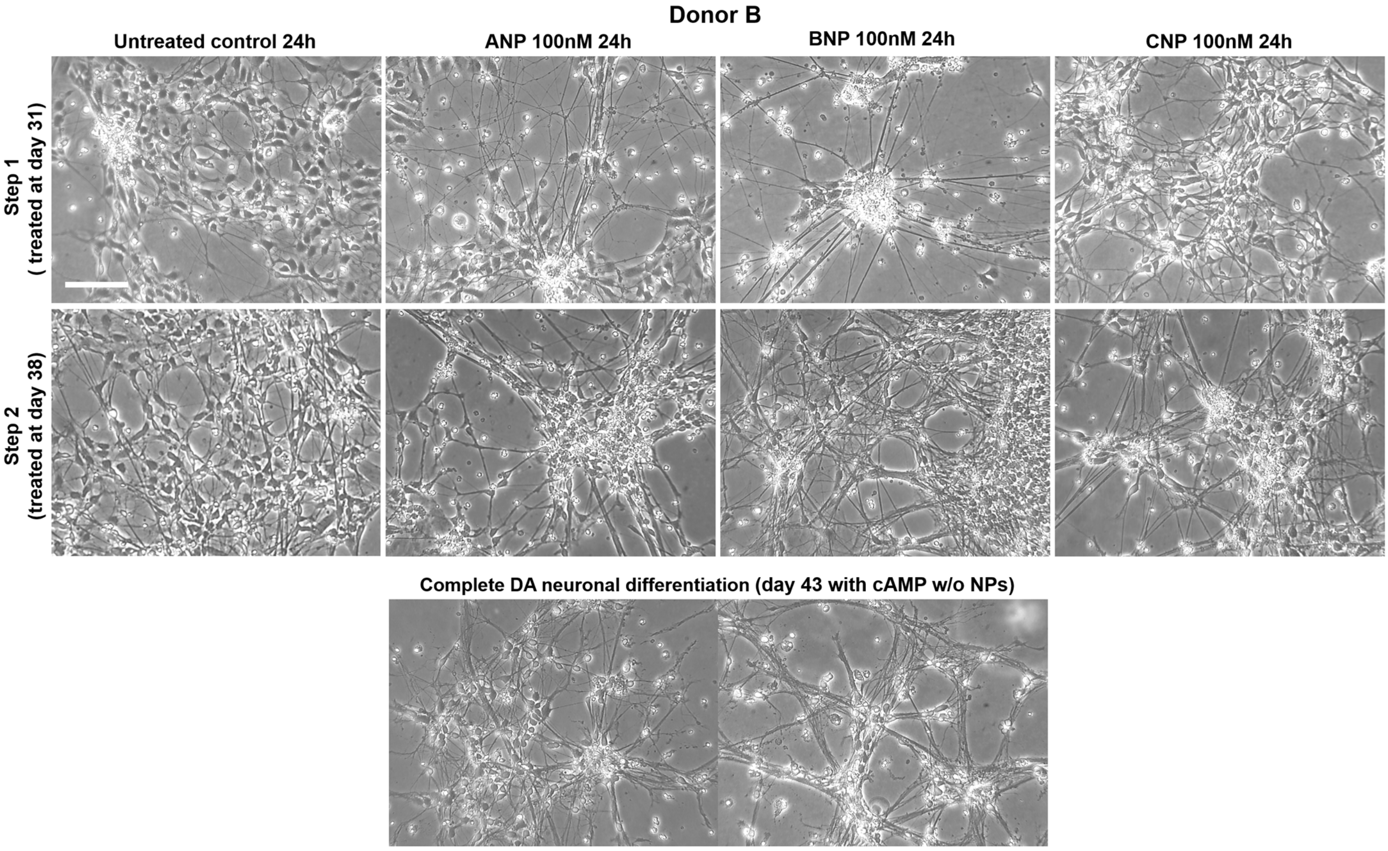

Supplementary Fig. S3. NPs enhance morphological features typical of completely differentiated DA neurons in hiPSCs-derived neuronal population. Phase contrast microscopy showing DA neuron-like morphology induced in hiPSCs from DONOR B by 24 h treatments with NPs performed at day 31 (Step 1) or at day 38 (Step 2), compared to complete dopaminergic neuronal differentiation obtained at day 43 in cultures maintained in NPs-free medium but in presence of cAMP. Bar 100 µm

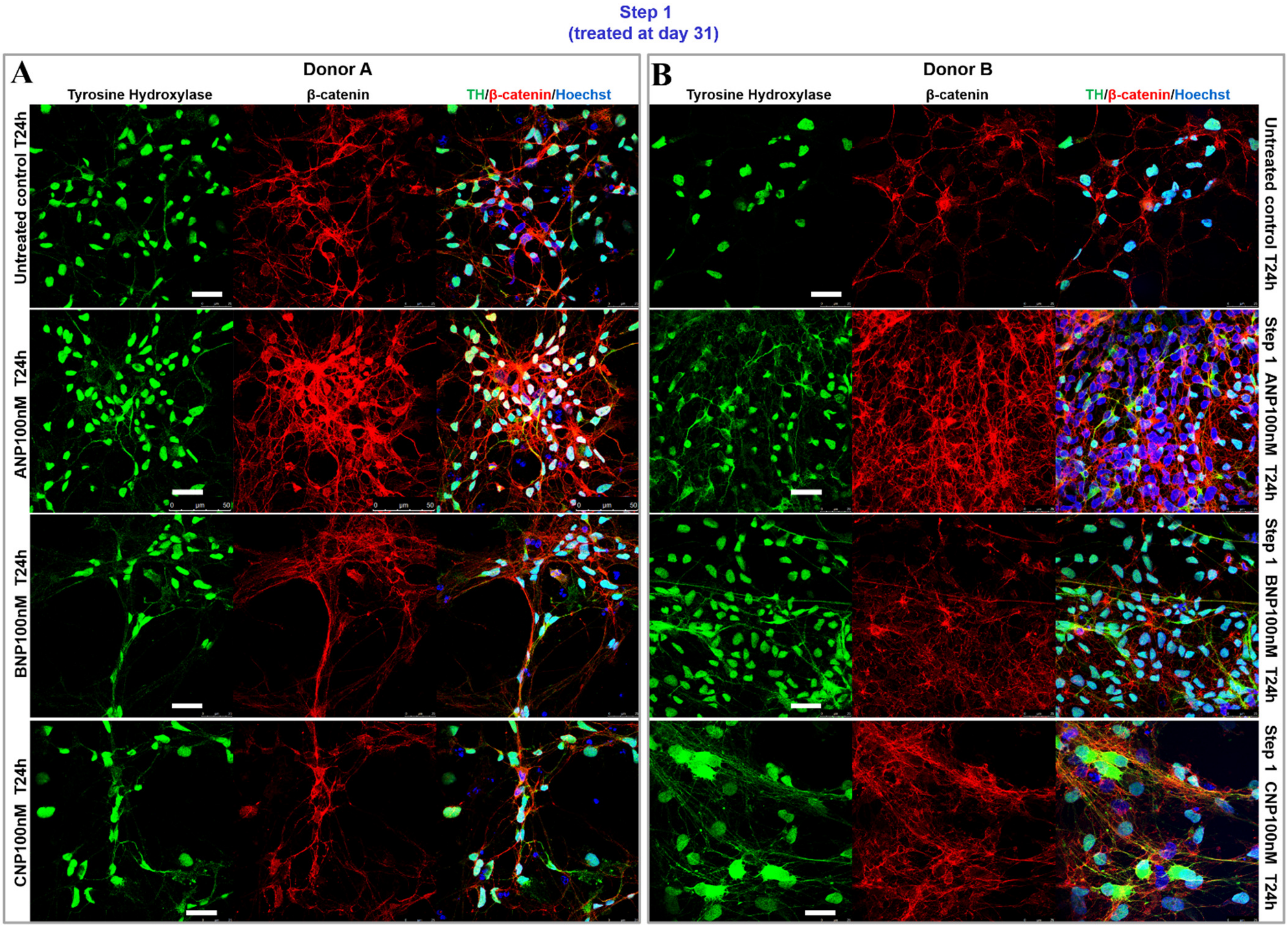

Supplementary Fig. S4. Comparative view of DA neuron differentiation induced by NPs treatments performed at day 31 (Step 1) on hiPSCs-derived neuronal population obtained from DONOR A (A) and from DONOR B (B). Confocal microscopy images showing the effects on the expression and intracellular distribution of TH (*green hue*) and β-catenin (*red hue*). Merged images of β-catenin/TH double immunofluorescent staining and nuclei counterstaining with Hoechst (*blue hue*) are also shown. Bars 25 μm

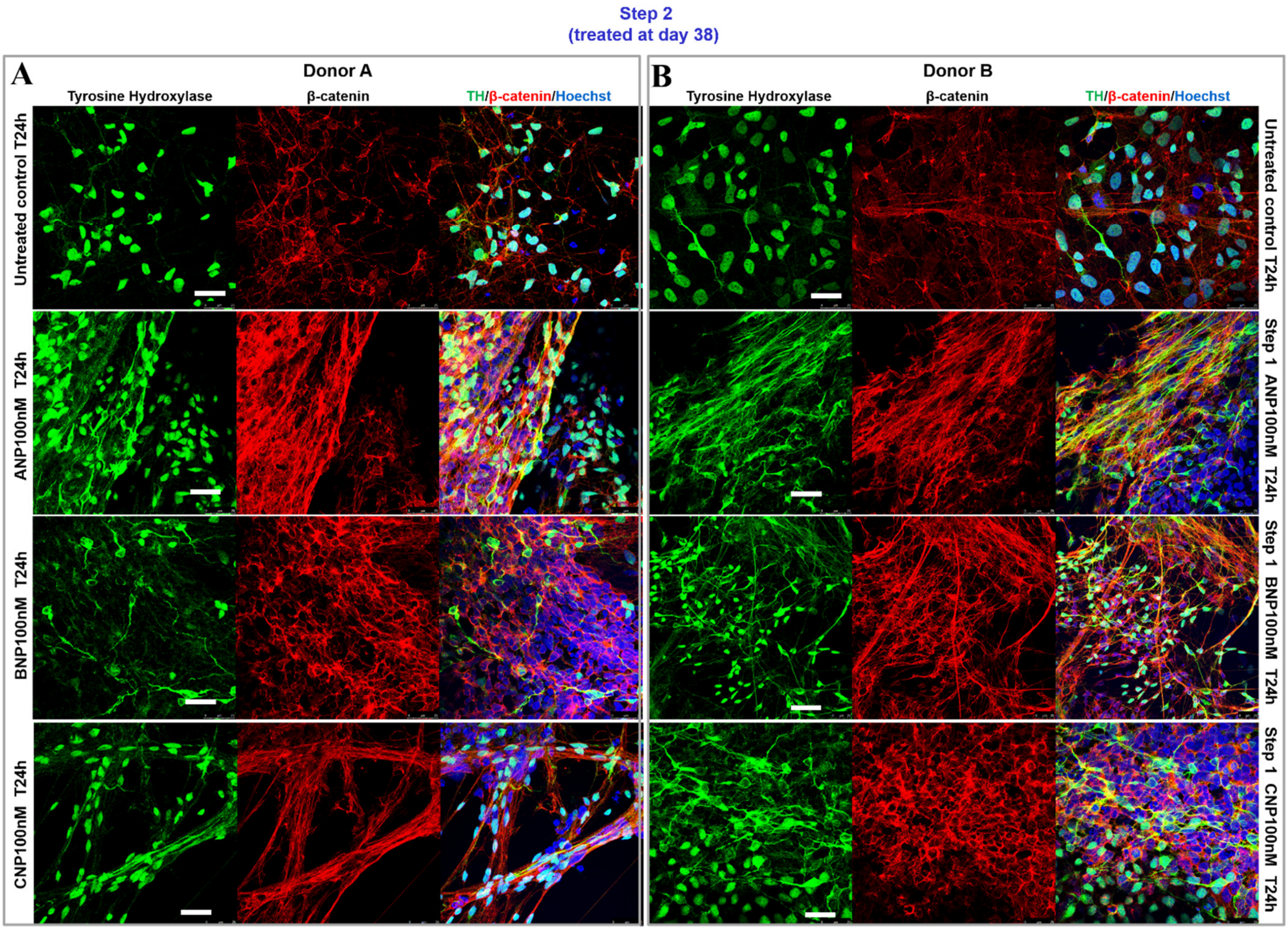

Supplementary Fig. S5. Comparative view of DA neuron differentiation induced by NPs treatments performed at day 38 (Step 2) on hiPSCs-derived neuronal population obtained from DONOR A (A) and from DONOR B (B). Confocal microscopy images showing the effects on the expression and intracellular distribution of TH (*green hue*) and β-catenin (*red hue*). Merged images of β-catenin/TH double immunofluorescent staining and nuclei counterstaining with Hoechst (*blue hue*) are also shown. Bars 25 μm

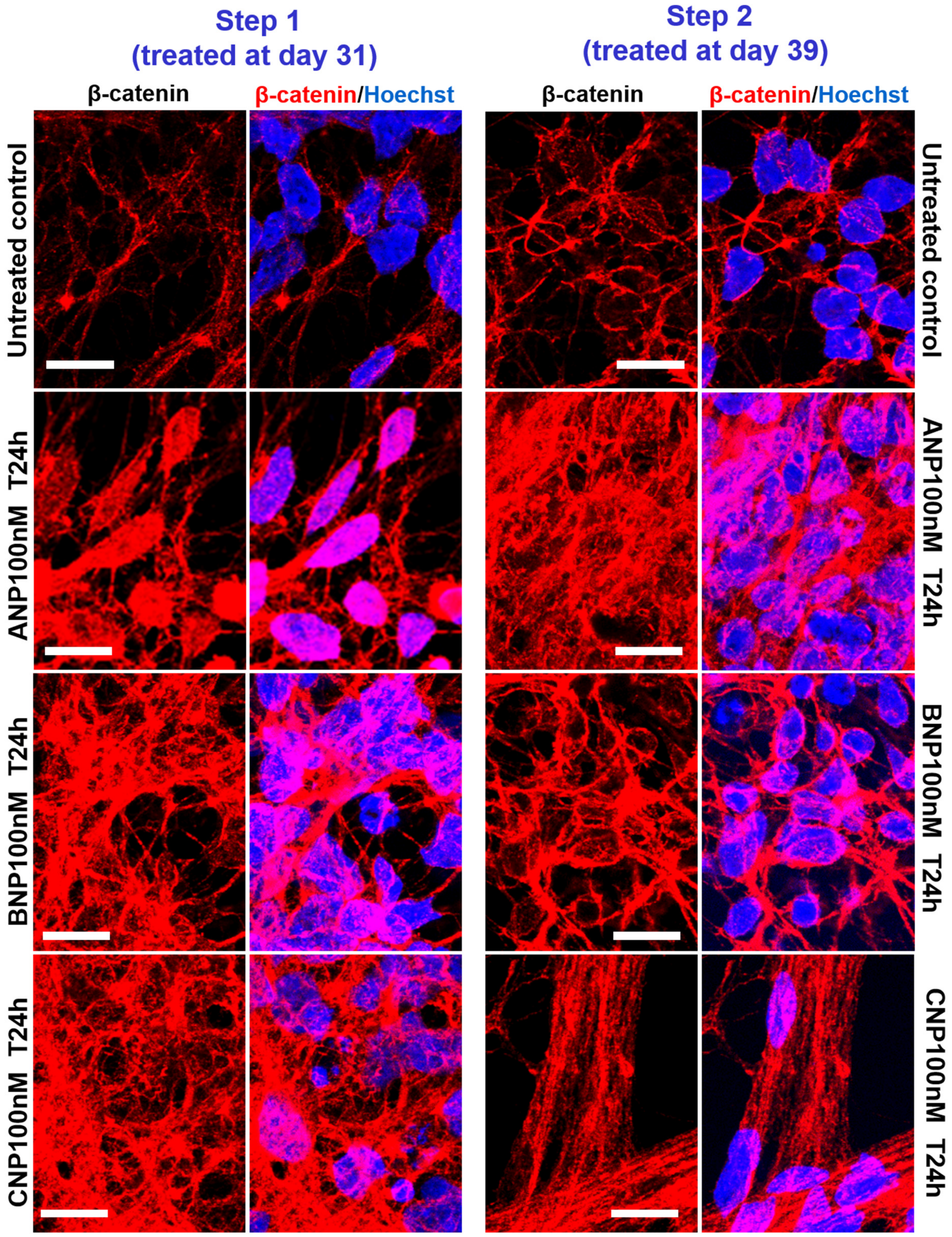

Supplementary Fig. S6. NPs-induced β-catenin cytoplasmic accumulation and nuclear translocation in hiPSCs-derived neuronal population from Donor A. Details at higher magnification of confocal microscopy images showing NPs effects on the intracellular distribution of β-catenin (*red hue*). Merged images of β-catenin and Hoechst nuclear staining (*blue hue*) are also shown. Bars 10 µm

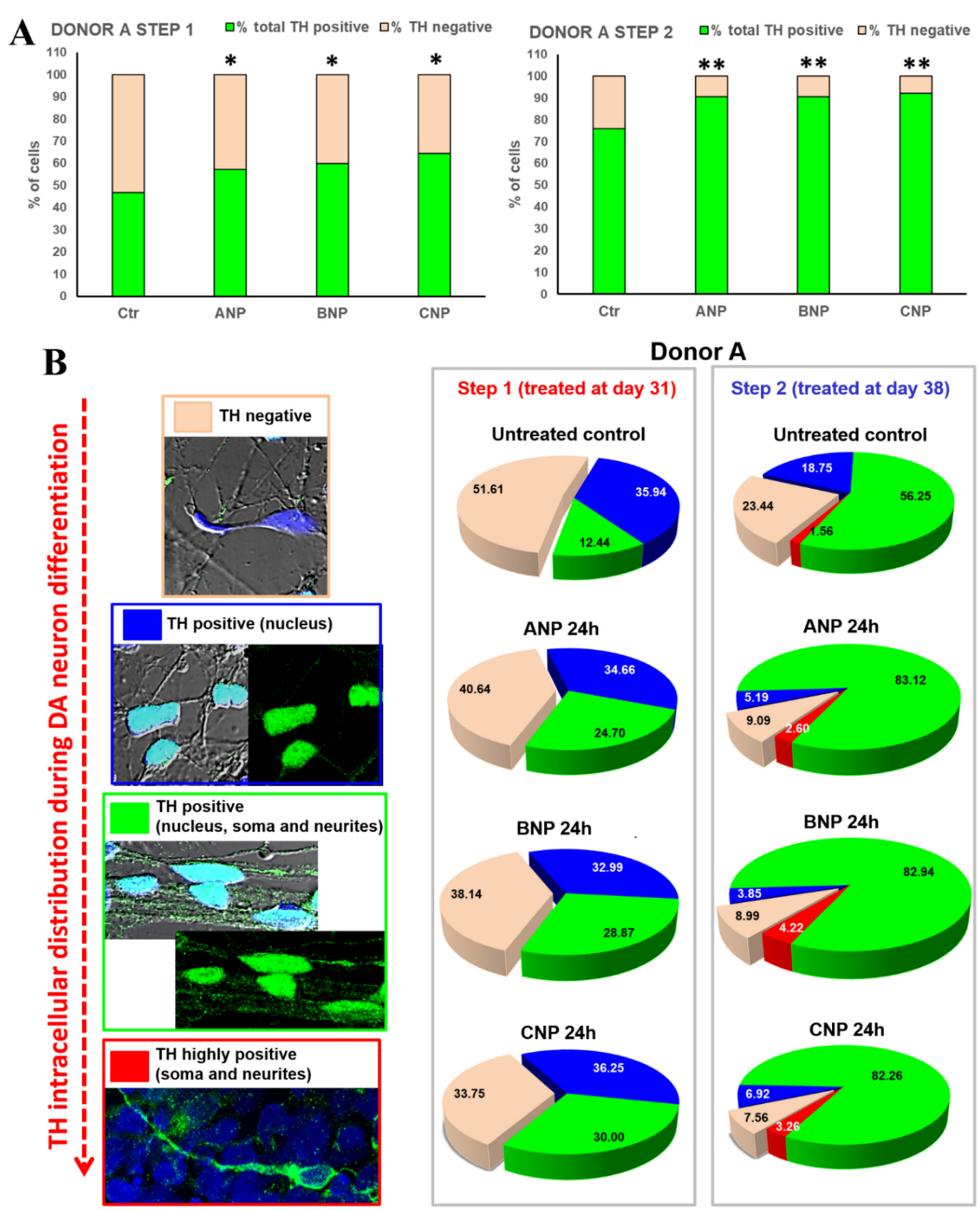

Supplementary Fig. S7. NPs increase the proportion of DA neurons expressing TH in the soma and along the axons and neurites: hiPSCs-derived neuronal population obtained from DONOR A. A Quantitative evaluation of percentage of TH^+ cells in cultures treated at day 31 (Step 1) or at day 38 (Step 2) with NPs, compared with the untreated controls. Data were obtained by counting a minimum of 300 cells/samples and results showed are the mean ± SD from three independent experiments ($n = 3$). Significance *vs* untreated control (two-tailed Student's t test): $*p < 0.05$. **B** *Left panels*: representative images by confocal microscopy showing the modification of the intracellular distribution of total TH (*green hue*) observed during NPs-induced dopaminergic differentiation. *Right panels*: Quantitative evaluation of NPs effect on the percentage of TH^- cells and of TH^+ neurons exhibiting this enzyme exclusively in the nucleus (poorly differentiated), in nucleus, soma and neurites (early differentiated) or exclusively in soma and neurites (mature DA neurons).

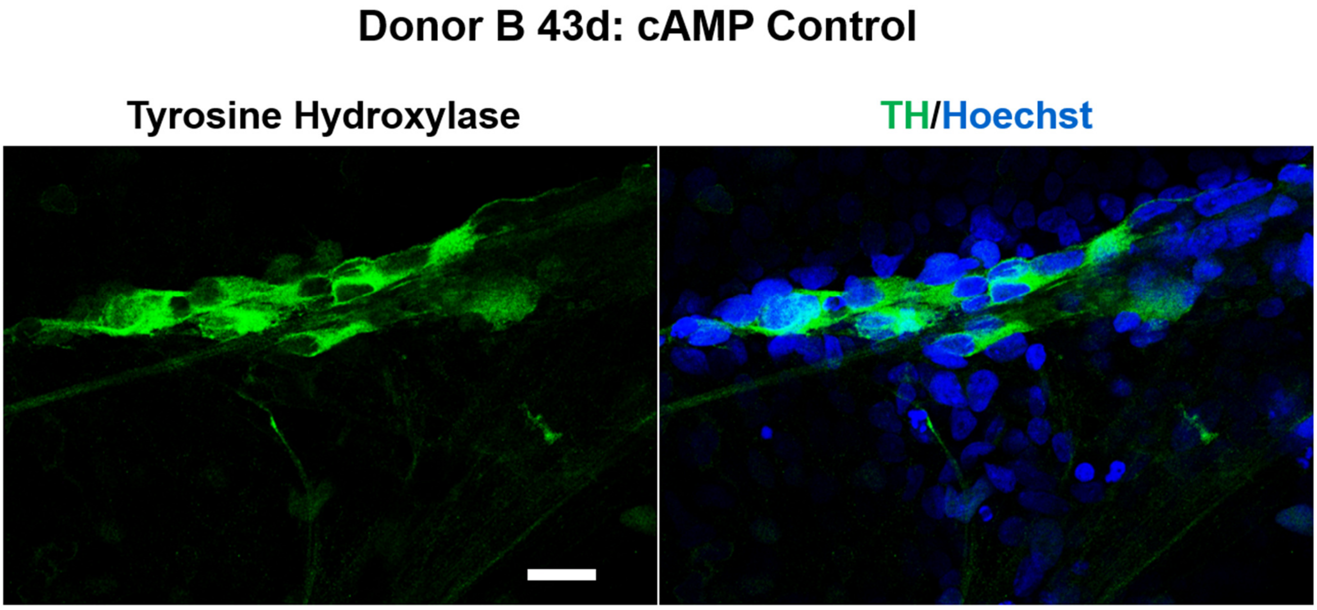

Supplementary Fig. S8. Tyrosine Hydroxylase intracellular distribution in cAMP-treated positive control of dopaminergic neurons from Donor B at 43 day. Confocal microscopy images showing that TH (green hue) was mainly, but not exclusively, localized in the cytoplasm. Merged image of TH and Hoechst nuclear staining (blue hue) is also shown. Bar 20 µm

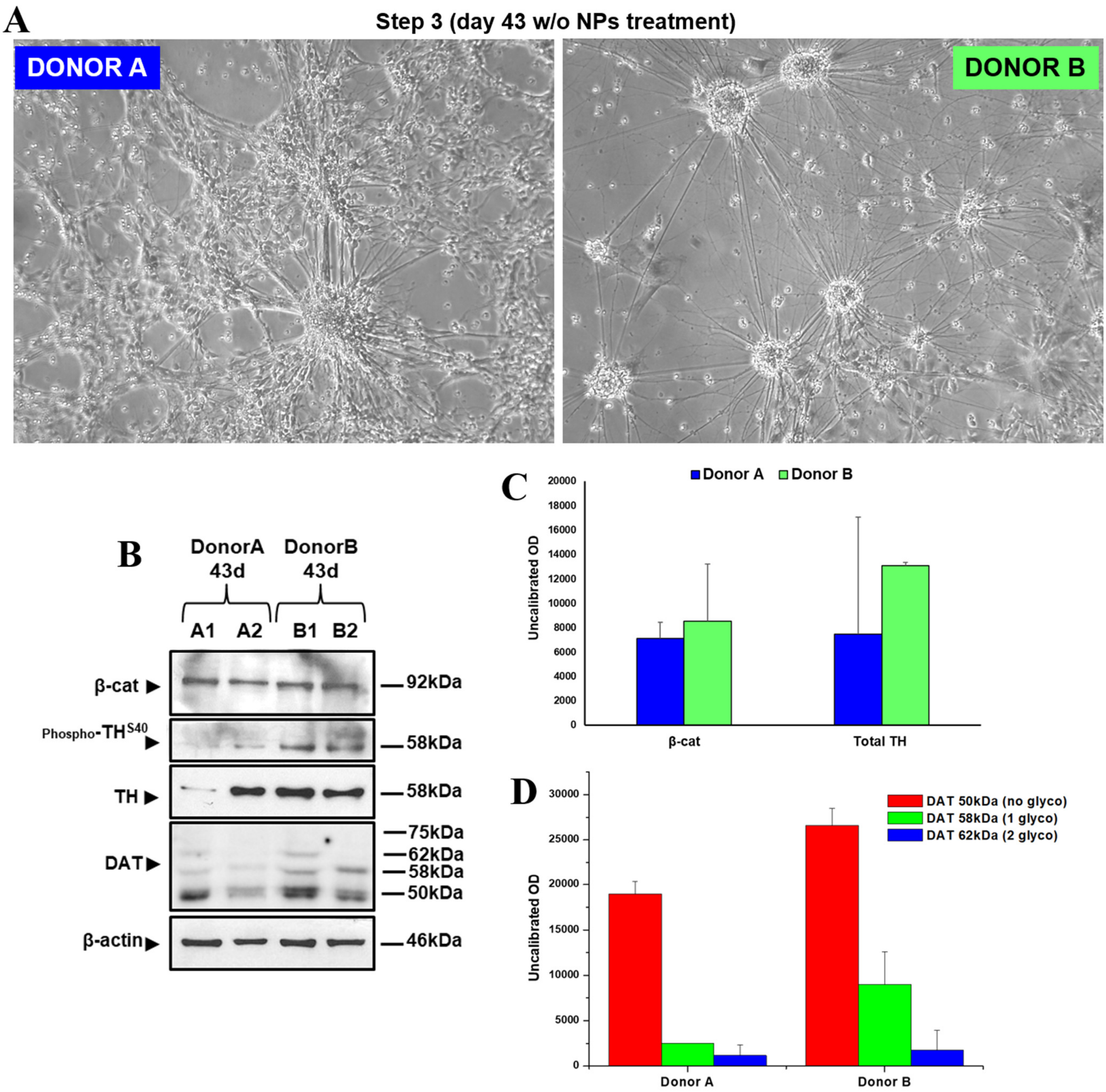

Supplementary Fig. S9. Characterization of hiPSCs-derived DA neuronal population from DONOR A and DONOR B showing the intrinsic variability of the hiPSCs lines from each donor. Comparative analyses of morphology (**A**), and of expression of β-catenin, TH and unglycosylated and glycosylated forms of DAT (**B**), in DA neurons after 43 days of differentiation in NPs-free medium. Lanes A1, A2 and B1, B2 in panel **B** report the WB results from two duplicates of the same batch of neural induction from Donor A (A1, A2) and form Donor B (B1 and B2), respectively. **C, D** Densitometric analysis of the WB showed in panel B, reported as mean values of uncalibrated optical density + SD obtained from A1-A2 and B1-B2 duplicates. The results from WB show that the neural population from donor B is more homogeneously differentiated than that from donor A, since the duplicates B1 and B2 exhibited comparable expression of TH, p-TH and DAT (expression/glycosylation), while the duplicates A1 and A2 do not.
