# Supplementary material for: Natriuretic peptides are neuroprotective on in vitro models of PD and promote dopaminergic differentiation of hiPSCs-derived neurons via the Wnt/β-catenin signaling
Source: Cell Death Discov. 2021 Nov 1;7:330. doi: 10.1038/s41420-021-00723-6 (PMC8560781; doi:10.1038/s41420-021-00723-6)
Supplement: Supplementary file 4 — Supplementary Data [file 41420_2021_723_MOESM4_ESM.pdf]

Fig 1 D   *P<0.05; **P<0.01; ***P<0.001; ****P<0.0001   Cellular model: SHSY5Y cell line

	β-catenin					
	Tukey's Multiple Comparison Test	Mean Diff.	q	Significant? P < 0.05?	Summary	95% CI of diff
6h of treatment	Ctr 6h vs ANP 6h	-1.92	53.44	Yes	***	-2.128 to -1.711
	Ctr 6h vs BNP 6h	-2.28	63.46	Yes	***	-2.489 to -2.071
	Ctr 6h vs CNP 6h	-2.759	76.81	Yes	***	-2.968 to -2.550
	ANP 6h vs BNP 6h	-0.3602	10.03	Yes	**	-0.5690 to -0.1513
	ANP 6h vs CNP 6h	-0.8396	23.37	Yes	***	-1.049 to -0.6307
	BNP 6h vs CNP 6h	-0.4795	13.35	Yes	***	-0.6884 to -0.2706
24h of treatment	Ctr 24h vs ANP 24h	-2.109	58.71	Yes	***	-2.318 to -1.900
	Ctr 24h vs BNP 24h	-1.561	43.45	Yes	***	-1.770 to -1.352
	Ctr 24h vs CNP 24h	-0.5232	14.56	Yes	***	-0.7321 to -0.3143
	ANP 24h vs BNP 24h	0.548	15.25	Yes	***	0.3391 to 0.7569
	ANP 24h vs CNP 24h	1.586	44.14	Yes	***	1.377 to 1.795
	BNP 24h vs CNP 24h	1.038	28.89	Yes	***	0.8289 to 1.247
Time-dependent effect	ANP 6h vs ANP 24h	-0.1894	5.272	No	ns	-0.3983 to 0.01949
	BNP 6h vs BNP 24h	0.7187	20.01	Yes	***	0.5098 to 0.9276
	CNP 6h vs CNP 24h	2.236	62.24	Yes	***	2.027 to 2.445

	phospho β-catenin					
	Tukey's Multiple Comparison Test	Mean Diff.	q	Significant? P < 0.05?	Summary	95% CI of diff
6h of treatment	Ctr 6h vs ANP 6h	-1.508	65.17	Yes	***	-1.642 to -1.373
	Ctr 6h vs BNP 6h	-1.045	45.17	Yes	***	-1.180 to -0.9106
	Ctr 6h vs CNP 6h	-0.7472	32.3	Yes	***	-0.8817 to -0.6127
	ANP 6h vs BNP 6h	0.4627	20	Yes	***	0.3281 to 0.5972
	ANP 6h vs CNP 6h	0.7606	32.87	Yes	***	0.6260 to 0.8951
	BNP 6h vs CNP 6h	0.2979	12.88	Yes	***	0.1634 to 0.4324
24h of treatment	Ctr 24h vs ANP 24h	-0.02438	1.054	No	ns	-0.1589 to 0.1102
	Ctr 24h vs BNP 24h	0.01912	0.827	No	ns	-0.1154 to 0.1537
	Ctr 24h vs CNP 24h	-1.026	44.34	Yes	***	-1.160 to -0.8913
	ANP 24h vs BNP 24h	0.0435	1.88	No	ns	-0.09103 to 0.1780
	ANP 24h vs CNP 24h	-1.001	43.29	Yes	***	-1.136 to -0.8669
	BNP 24h vs CNP 24h	-1.045	45.17	Yes	***	-1.179 to -0.9104

**Time-dependent effect**	ANP 6h vs ANP 24h	1.483	64.12	Yes	***	1.349 to 1.618
	BNP 6h vs BNP 24h	1.064	46	Yes	***	0.9297 to 1.199
	CNP 6h vs CNP 24h	-0.2786	12.04	Yes	***	-0.4132 to -0.1441

	**GSK**					
	**Tukey's Multiple Comparison Test**	**Mean Diff.**	**q**	**Significant? P < 0.05?**	**Summary**	**95% CI of diff**
**6h of treatment**	Ctr 6h vs ANP 6h	-2.805	82.48	Yes	***	-3.003 to -2.607
	Ctr 6h vs BNP 6h	-1.922	56.53	Yes	***	-2.120 to -1.725
	Ctr 6h vs CNP 6h	-2.14	62.92	Yes	***	-2.337 to -1.942
	ANP 6h vs BNP 6h	0.8824	25.95	Yes	***	0.6847 to 1.080
	ANP 6h vs CNP 6h	0.6652	19.56	Yes	***	0.4675 to 0.8630
	BNP 6h vs CNP 6h	-0.2172	6.387	Yes	*	-0.4149 to -0.01943
**24h of treatment**	Ctr 24h vs ANP 24h	-0.8225	24.19	Yes	***	-1.020 to -0.6248
	Ctr 24h vs BNP 24h	-1.236	36.34	Yes	***	-1.433 to -1.038
	Ctr 24h vs CNP 24h	-0.6291	18.5	Yes	***	-0.8268 to -0.4313
	ANP 24h vs BNP 24h	-0.4131	12.15	Yes	***	-0.6109 to -0.2154
	ANP 24h vs CNP 24h	0.1935	5.69	No	**ns**	-0.004267 to 0.3912
	BNP 24h vs CNP 24h	0.6066	17.84	Yes	***	0.4089 to 0.8043
**Time-dependent effect**	ANP 6h vs ANP 24h	1.982	58.29	Yes	***	1.785 to 2.180
	BNP 6h vs BNP 24h	0.6867	20.19	Yes	***	0.4890 to 0.8844
	CNP 6h vs CNP 24h	1.51	44.42	Yes	***	1.313 to 1.708

	**phospho GSK**					
	**Tukey's Multiple Comparison Test**	**Mean Diff.**	**q**	**Significant? P < 0.05?**	**Summary**	**95% CI of diff**
**6h of treatment**	Ctr 6h vs ANP 6h	-0.2652	10.3	Yes	**	-0.4149 to -0.1155
	Ctr 6h vs BNP 6h	-0.4451	17.29	Yes	***	-0.5948 to -0.2954
	Ctr 6h vs CNP 6h	-0.4177	16.22	Yes	***	-0.5674 to -0.2680
	ANP 6h vs BNP 6h	-0.1799	6.988	Yes	*	-0.3296 to -0.03019
	ANP 6h vs CNP 6h	-0.1525	5.922	Yes	*	-0.3022 to -0.002764
	BNP 6h vs CNP 6h	0.02743	1.065	No	**ns**	-0.1223 to 0.1771
**24h of treatment**	Ctr 24h vs ANP 24h	-0.2493	9.684	Yes	**	-0.3990 to -0.09961
	Ctr 24h vs BNP 24h	-0.07142	2.774	No	**ns**	-0.2211 to 0.07829
	Ctr 24h vs CNP 24h	-1.269	49.31	Yes	***	-1.419 to -1.120
	ANP 24h vs BNP 24h	0.1779	6.91	Yes	*	0.02819 to 0.3276

	ANP 24h vs CNP 24h	-1.02	39.63	Yes	***	-1.170 to -0.8704
	BNP 24h vs CNP 24h	-1.198	46.54	Yes	***	-1.348 to -1.048
**Time-dependent effect**	ANP 6h vs ANP 24h	0.01588	0.617	No	**ns**	-0.1338 to 0.1656
	BNP 6h vs BNP 24h	0.3737	14.51	Yes	***	0.2240 to 0.5234
	CNP 6h vs CNP 24h	-0.8518	33.09	Yes	***	-1.002 to -0.7021

Fig 1 E ***P<0.05; **P<0.01; ***P<0.001; ****P<0.0001**

	**Tyrosine hidroxylase (TH)**					
	**Tukey's Multiple Comparison Test**	**Mean Diff.**	**q**	**Significant? P < 0.05?**	**Summary**	**95% CI of diff**
**6h of treatment**	Ctr 6h vs ANP 6h	-3.532	62.83	Yes	***	-3.859 to -3.205
	Ctr 6h vs BNP 6h	-2.847	50.65	Yes	***	-3.174 to -2.521
	Ctr 6h vs CNP 6h	-4.573	81.34	Yes	***	-4.900 to -4.246
	ANP 6h vs BNP 6h	0.6846	12.18	Yes	***	0.3577 to 1.011
	ANP 6h vs CNP 6h	-1.041	18.52	Yes	***	-1.368 to -0.7140
	BNP 6h vs CNP 6h	-1.725	30.69	Yes	***	-2.052 to -1.399
**24h of treatment**	Ctr 24h vs ANP 24h	-4.757	84.62	Yes	***	-5.084 to -4.430
	Ctr 24h vs BNP 24h	-2.773	49.34	Yes	***	-3.100 to -2.447
	Ctr 24h vs CNP 24h	-3.934	69.98	Yes	***	-4.261 to -3.607
	ANP 24h vs BNP 24h	1.984	35.29	Yes	***	1.657 to 2.311
	ANP 24h vs CNP 24h	0.8234	14.65	Yes	***	0.4965 to 1.150
	BNP 24h vs CNP 24h	-1.16	20.64	Yes	***	-1.487 to -0.8335
**Time-dependent effect**	ANP 6h vs ANP 24h	-1.225	21.8	Yes	***	-1.552 to -0.8984
	BNP 6h vs BNP 24h	0.07399	1.316	No	**ns**	-0.2529 to 0.4009
	CNP 6h vs CNP 24h	0.6391	11.37	Yes	**	0.3122 to 0.9660

	**DJ-1**					
	**Tukey's Multiple Comparison Test**	**Mean Diff.**	**q**	**Significant? P < 0.05?**	**Summary**	**95% CI of diff**
**6h of treatment**	Ctr 6h vs ANP 6h	-1.732	57.39	Yes	***	-1.907 to -1.556
	Ctr 6h vs BNP 6h	-1.794	59.45	Yes	***	-1.969 to -1.618
	Ctr 6h vs CNP 6h	-1.358	45	Yes	***	-1.533 to -1.182
	ANP 6h vs BNP 6h	-0.0622	2.061	No	**ns**	-0.2377 to 0.1133
	ANP 6h vs CNP 6h	0.3738	12.39	Yes	***	0.1984 to 0.5493
	BNP 6h vs CNP 6h	0.436	14.45	Yes	***	0.2606 to 0.6115

**24h of treatment**	Ctr 24h vs ANP 24h	-1.751	58.02	Yes	***	-1.926 to -1.575
	Ctr 24h vs BNP 24h	-1.916	63.48	Yes	***	-2.091 to -1.740
	Ctr 24h vs CNP 24h	-0.7018	23.26	Yes	***	-0.8773 to -0.5264
	ANP 24h vs BNP 24h	-0.1646	5.456	No	**ns**	-0.3401 to 0.01083
	ANP 24h vs CNP 24h	1.049	34.76	Yes	***	0.8735 to 1.224
	BNP 24h vs CNP 24h	1.214	40.22	Yes	***	1.038 to 1.389
**Time-dependent effect**	ANP 6h vs ANP 24h	-0.01908	0.632	No	**ns**	-0.1946 to 0.1564
	BNP 6h vs BNP 24h	-0.1215	4.027	No	**ns**	-0.2970 to 0.05394
	CNP 6h vs CNP 24h	0.6561	21.74	Yes	***	0.4806 to 0.8316

	**Nurr1**					
	**Tukey's Multiple Comparison Test**	**Mean Diff.**	**q**	**Significant? P < 0.05?**	**Summary**	**95% CI of diff**
**6h of treatment**	Ctr 6h vs ANP 6h	-1.018	32.34	Yes	***	-1.201 to -0.8351
	Ctr 6h vs BNP 6h	-1.368	43.46	Yes	***	-1.551 to -1.185
	Ctr 6h vs CNP 6h	-1.764	56.02	Yes	***	-1.947 to -1.580
	ANP 6h vs BNP 6h	-0.35	11.12	Yes	**	-0.5330 to -0.1669
	ANP 6h vs CNP 6h	-0.7454	23.68	Yes	***	-0.9285 to -0.5623
	BNP 6h vs CNP 6h	-0.3954	12.56	Yes	***	-0.5785 to -0.2124
**24h of treatment**	Ctr 24h vs ANP 24h	-2.118	67.29	Yes	***	-2.302 to -1.935
	Ctr 24h vs BNP 24h	-2.306	73.24	Yes	***	-2.489 to -2.123
	Ctr 24h vs CNP 24h	-1.065	33.82	Yes	***	-1.248 to -0.8818
	ANP 24h vs BNP 24h	-0.1872	5.946	Yes	*	-0.3703 to -0.004128
	ANP 24h vs CNP 24h	1.054	33.47	Yes	***	0.8705 to 1.237
	BNP 24h vs CNP 24h	1.241	39.41	Yes	***	1.058 to 1.424
**Time-dependent effect**	ANP 6h vs ANP 24h	-1.1	34.95	Yes	***	-1.283 to -0.9172
	BNP 6h vs BNP 24h	-0.9375	29.78	Yes	***	-1.121 to -0.7545
	CNP 6h vs CNP 24h	0.6987	22.19	Yes	***	0.5157 to 0.8818

.

Fig 2B	*P<0.05; **P<0.01; ***P<0.001; ****P<0.0001		Cellular model: SHSY5Y cell line			
	Neuroprotection in SHSY5Y cells (cytotoxicity)					
	Tukey's Multiple Comparison Test	Mean Diff.	q	Significant? P < 0.05?	Summary	95% CI of diff
Protection from neurotoxin induced stress	CTR vs 6-OHDA	21.7	20.68	Yes	***	15.11 to 28.30
	CTR vs preANP 24h + 6-OHDA	-0.9453	0.9009	No	ns	-7.542 to 5.651
	CTR vs preBNP 24h + 6-OHDA	-0.661	0.63	No	ns	-7.258 to 5.936
	CTR vs preCNP 24h + 6-OHDA	6.368	6.069	No	ns	-0.2290 to 12.96
	6-OHDA vs preANP 24h + 6-OHDA	-22.65	21.58	Yes	***	-29.25 to -16.05
	6-OHDA vs preBNP 24h + 6-OHDA	-22.36	21.31	Yes	***	-28.96 to -15.77
	6-OHDA vs preCNP 24h + 6-OHDA	-15.34	14.62	Yes	**	-21.93 to -8.738
Comparative efficacy in neuroprotection	preANP 24h + 6-OHDA vs preBNP 24h + 6-OHDA	0.2843	0.271	No	ns	-6.312 to 6.881
	preANP 24h + 6-OHDA vs preCNP 24h + 6-OHDA	7.313	6.97	Yes	*	0.7163 to 13.91
	preBNP 24h + 6-OHDA vs preCNP 24h + 6-OHDA	7.029	6.699	Yes	*	0.4320 to 13.63

Fig 4B	*P<0.05; **P<0.01; ***P<0.001; ****P<0.0001		Cellular model: primary cultures of DA neurons			
	Nuclear β-catenin in primary DA neurons					
	Tukey's Multiple Comparison Test	Mean Diff.	q	Significant? P < 0.05?	Summary	95% CI of diff
Efficacy in β-cat nuclear translocation	Ctr vs ANP	-10.97	65.27	Yes	***	-12.11 to -9.819
	Ctr vs BNP	-12.53	74.61	Yes	***	-13.68 to -11.39
	Ctr vs CNP	-23.04	137.2	Yes	***	-24.19 to -21.90
Comparative efficacy in β-cat nuclear translocation	ANP vs BNP	-1.569	9.337	Yes	*	-2.715 to -0.4221
	ANP vs CNP	-12.08	71.89	Yes	***	-13.22 to -10.93
	BNP vs CNP	-10.51	62.55	Yes	***	-11.66 to -9.363

Fig 4D	*P<0.05; **P<0.01; ***P<0.001; ****P<0.0001		Cellular model: primary cultures of DA neurons			
	Neuroprotection in primary DA neurons (% of TH^{+} cells)					
	Tukey's Multiple Comparison Test	Mean Diff.	q	Significant? P < 0.05?	Summary	95% CI of diff
Peptide neurotoxicity	Ctr vs ANP	-2.664	1.954	No	ns	-10.59 to 5.263
	Ctr vs BNP	-2.723	1.998	No	ns	-10.65 to 5.204
	Ctr vs CNP	-2.476	1.816	No	ns	-10.40 to 5.452
Protection from neurotoxin induced stress	Ctr vs 6OH-DA	73.27	53.74	Yes	***	65.34 to 81.19
	ANP vs 6OH-DA	75.93	55.7	Yes	***	68.00 to 83.86
	BNP vs 6OH-DA	75.99	55.74	Yes	***	68.06 to 83.92
	CNP vs 6OH-DA	75.74	55.56	Yes	***	67.81 to 83.67
	6OH-DA vs preANP+6OH	-16.44	12.06	Yes	***	-24.37 to -8.517
	6OH-DA vs preBNP+6OH	-19.91	14.61	Yes	***	-27.84 to -11.99
	6OH-DA vs preCNP+6OH	-55.38	40.62	Yes	***	-63.30 to -47.45
Comparative efficacy in neuroprotection	preANP+6OH vs preBNP+6OH	-3.469	2.545	No	ns	-11.40 to 4.458
	preANP+6OH vs preCNP+6OH	-38.93	28.56	Yes	***	-46.86 to -31.01
	preBNP+6OH vs preCNP+6OH	-35.46	26.02	Yes	***	-43.39 to -27.54

Fig 2B	*P<0.05; **P<0.01; ***P<0.001; ****P<0.0001		Cellular model: SHSY5Y cell line			
	Neuroprotection in SHSY5Y cells (cytotoxicity)					
	Tukey's Multiple Comparison Test	Mean Diff.	q	Significant? P < 0.05?	Summary	95% CI of diff
Protection from neurotoxin induced stress	CTR vs 6-OHDA	21.7	20.68	Yes	***	15.11 to 28.30
	CTR vs preANP 24h + 6-OHDA	-0.9453	0.9009	No	ns	-7.542 to 5.651
	CTR vs preBNP 24h + 6-OHDA	-0.661	0.63	No	ns	-7.258 to 5.936
	CTR vs preCNP 24h + 6-OHDA	6.368	6.069	No	ns	-0.2290 to 12.96
	6-OHDA vs preANP 24h + 6-OHDA	-22.65	21.58	Yes	***	-29.25 to -16.05
	6-OHDA vs preBNP 24h + 6-OHDA	-22.36	21.31	Yes	***	-28.96 to -15.77
	6-OHDA vs preCNP 24h + 6-OHDA	-15.34	14.62	Yes	**	-21.93 to -8.738
Comparative efficacy in neuroprotection	preANP 24h + 6-OHDA vs preBNP 24h + 6-OHDA	0.2843	0.271	No	ns	-6.312 to 6.881
	preANP 24h + 6-OHDA vs preCNP 24h + 6-OHDA	7.313	6.97	Yes	*	0.7163 to 13.91
	preBNP 24h + 6-OHDA vs preCNP 24h + 6-OHDA	7.029	6.699	Yes	*	0.4320 to 13.63

Fig 4B	*P<0.05; **P<0.01; ***P<0.001; ****P<0.0001		Cellular model: primary cultures of DA neurons			
	Nuclear β-catenin in primary DA neurons					
	Tukey's Multiple Comparison Test	Mean Diff.	q	Significant? P < 0.05?	Summary	95% CI of diff
Efficacy in β-cat nuclear translocation	Ctr vs ANP	-10.97	65.27	Yes	***	-12.11 to -9.819
	Ctr vs BNP	-12.53	74.61	Yes	***	-13.68 to -11.39
	Ctr vs CNP	-23.04	137.2	Yes	***	-24.19 to -21.90
Comparative efficacy in β-cat nuclear translocation	ANP vs BNP	-1.569	9.337	Yes	*	-2.715 to -0.4221
	ANP vs CNP	-12.08	71.89	Yes	***	-13.22 to -10.93
	BNP vs CNP	-10.51	62.55	Yes	***	-11.66 to -9.363

Fig 4D	*P<0.05; **P<0.01; ***P<0.001; ****P<0.0001		Cellular model: primary cultures of DA neurons			
	Neuroprotection in primary DA neurons (% of TH^+ cells)					
	Tukey's Multiple Comparison Test	Mean Diff.	q	Significant? P < 0.05?	Summary	95% CI of diff
Peptide neurotoxicity	Ctr vs ANP	-2.664	1.954	No	ns	-10.59 to 5.263
	Ctr vs BNP	-2.723	1.998	No	ns	-10.65 to 5.204
	Ctr vs CNP	-2.476	1.816	No	ns	-10.40 to 5.452
Protection from neurotoxin induced stress	Ctr vs 6OH-DA	73.27	53.74	Yes	***	65.34 to 81.19
	ANP vs 6OH-DA	75.93	55.7	Yes	***	68.00 to 83.86
	BNP vs 6OH-DA	75.99	55.74	Yes	***	68.06 to 83.92
	CNP vs 6OH-DA	75.74	55.56	Yes	***	67.81 to 83.67
	6OH-DA vs preANP+6OH	-16.44	12.06	Yes	***	-24.37 to -8.517
	6OH-DA vs preBNP+6OH	-19.91	14.61	Yes	***	-27.84 to -11.99
	6OH-DA vs preCNP+6OH	-55.38	40.62	Yes	***	-63.30 to -47.45
Comparative efficacy in neuroprotection	preANP+6OH vs preBNP+6OH	-3.469	2.545	No	ns	-11.40 to 4.458
	preANP+6OH vs preCNP+6OH	-38.93	28.56	Yes	***	-46.86 to -31.01
	preBNP+6OH vs preCNP+6OH	-35.46	26.02	Yes	***	-43.39 to -27.54

Fig 6C, D | *P<0.05; **P<0.01; ***P<0.001; ****P<0.0001 | Cellular model: Human IPSCs from DONOR A

	β-catenin					
	Tukey's Multiple Comparison Test	Mean Diff.	q	Significant? P < 0.05?	Summary	95% CI of diff
Treatment at 31 days	Ctr Step 1 vs ANP Step 1	-1.71	37.57	Yes	***	-1.975 to -1.445
	Ctr Step 1 vs BNP Step 1	-1.329	29.2	Yes	***	-1.594 to -1.065
	Ctr Step 1 vs CNP Step 1	-0.9063	19.91	Yes	***	-1.171 to -0.6417
	ANP Step 1 vs BNP Step 1	0.3809	8.367	Yes	**	0.1162 to 0.6455
	ANP Step 1 vs CNP Step 1	0.8038	17.66	Yes	***	0.5391 to 1.069
	BNP Step 1 vs CNP Step 1	0.423	9.292	Yes	**	0.1583 to 0.6877
Treatment at 38 days	Ctr Step 2 vs ANP Step 2	-3.064	67.31	Yes	***	-3.328 to -2.799
	Ctr Step 2 vs BNP Step 2	-2.921	64.18	Yes	***	-3.186 to -2.656
	Ctr Step 2 vs CNP Step 2	-1.993	43.79	Yes	***	-2.258 to -1.728
	ANP Step 2 vs BNP Step 2	0.1424	3.128	No	ns	-0.1223 to 0.4071
	ANP Step 2 vs CNP Step 2	1.07	23.52	Yes	***	0.8058 to 1.335
	BNP Step 2 vs CNP Step 2	0.9281	20.39	Yes	***	0.6634 to 1.193

	Tyrosine hidroxylase (TH)					
	Tukey's Multiple Comparison Test	Mean Diff.	q	Significant? P < 0.05?	Summary	95% CI of diff
Treatment at 31 days	Ctr Step 1 vs ANP Step 1	0.265	4.762	No	ns	-0.007475 to 0.5374
	Ctr Step 1 vs BNP Step 1	0.01853	0.333	No	ns	-0.2539 to 0.2910
	Ctr Step 1 vs CNP Step 1	0.189	3.397	No	ns	-0.08343 to 0.4614
	ANP Step 1 vs BNP Step 1	-0.2464	4.429	No	ns	-0.5189 to 0.02600
	ANP Step 1 vs CNP Step 1	-0.07596	1.365	No	ns	-0.3484 to 0.1965
	BNP Step 1 vs CNP Step 1	0.1705	3.064	No	ns	-0.1020 to 0.4429
Treatment at 38 days	Ctr Step 2 vs ANP Step 2	0.6586	58.74	Yes	***	0.5821 to 0.7351
	Ctr Step 2 vs BNP Step 2	0.589	52.53	Yes	***	0.5125 to 0.6656

	Ctr Step 2 vs CNP Step 2	0.06822	6.085	No	ns	-0.008302 to 0.1447
	ANP Step 2 vs BNP Step 2	-0.0696	6.207	No	ns	-0.1461 to 0.006927
	ANP Step 2 vs CNP Step 2	-0.5904	52.66	Yes	***	-0.6669 to -0.5139
	BNP Step 2 vs CNP Step 2	-0.5208	46.45	Yes	***	-0.5973 to -0.4443

	**phospho TH**					
	**Tukey's Multiple Comparison Test**	**Mean Diff.**	**q**	**Significant? P < 0.05?**	**Summary**	**95% CI of diff**
**Treatment at 31 days**	Ctr Step 1 vs ANP Step 1	-1.627	19.63	Yes	***	-2.033 to -1.221
	Ctr Step 1 vs BNP Step 1	-0.3599	4.342	No	ns	-0.7658 to 0.04590
	Ctr Step 1 vs CNP Step 1	-0.0699	0.8432	No	ns	-0.4757 to 0.3359
	ANP Step 1 vs BNP Step 1	1.267	15.29	Yes	***	0.8613 to 1.673
	ANP Step 1 vs CNP Step 1	1.557	18.79	Yes	***	1.151 to 1.963
	BNP Step 1 vs CNP Step 1	0.29	3.499	No	ns	-0.1158 to 0.6959
**Treatment at 38 days**	Ctr Step 2 vs ANP Step 2	-0.5254	24.7	Yes	**	-0.6706 to -0.3802
	Ctr Step 2 vs BNP Step 2	-0.4649	21.86	Yes	**	-0.6101 to -0.3197
	Ctr Step 2 vs CNP Step 2	-0.3757	17.66	Yes	**	-0.5209 to -0.2305
	ANP Step 2 vs BNP Step 2	0.0605	2.844	No	ns	-0.08466 to 0.2057
	ANP Step 2 vs CNP Step 2	0.1497	7.038	Yes	*	0.004531 to 0.2949
	BNP Step 2 vs CNP Step 2	0.0892	4.194	No	ns	-0.05597 to 0.2344

	**DAT 50kDa (not glycosylated form)**					

	Tukey's Multiple Comparison Test	Mean Diff.	q	Significant? P < 0.05?	Summary	95% CI of diff
**Treatment at 31 days**	Ctr Step 1 vs ANP Step 1	1987	85.51	Yes	***	1829 to 2146
	Ctr Step 1 vs BNP Step 1	1706	73.41	Yes	***	1547 to 1865
	Ctr Step 1 vs CNP Step 1	416.7	17.93	Yes	**	258.1 to 575.3
	ANP Step 1 vs BNP Step 1	-281.2	12.1	Yes	*	-439.8 to -122.6
	ANP Step 1 vs CNP Step 1	-1571	67.58	Yes	***	-1729 to -1412
	BNP Step 1 vs CNP Step 1	-1289	55.48	Yes	***	-1448 to -1131
**Treatment at 38 days**	Ctr Step 2 vs ANP Step 2	-11172	53.04	Yes	***	-12610 to -9735
	Ctr Step 2 vs BNP Step 2	-4100	19.47	Yes	**	-5538 to -2663
	Ctr Step 2 vs CNP Step 2	297.7	1.413	No	**ns**	-1140 to 1735
	ANP Step 2 vs BNP Step 2	7072	33.58	Yes	***	5634 to 8510
	ANP Step 2 vs CNP Step 2	11470	54.46	Yes	***	10033 to 12908
	BNP Step 2 vs CNP Step 2	4398	20.88	Yes	**	2961 to 5836
	**DAT 58kDa (mono-glycosylated form)**					
	**Tukey's Multiple Comparison Test**	**Mean Diff.**	**q**	**Significant? P < 0.05?**	**Summary**	**95% CI of diff**
**Treatment at 31 days**	Ctr Step 1 vs ANP Step 1	-0.337	0.8386	No	**ns**	-3.080 to 2.406
	Ctr Step 1 vs BNP Step 1	0.0385	0.0958	No	**ns**	-2.704 to 2.781
	Ctr Step 1 vs CNP Step 1	0.899	2.237	No	**ns**	-1.844 to 3.642
	ANP Step 1 vs BNP Step 1	0.3755	0.9344	No	**ns**	-2.367 to 3.118
	ANP Step 1 vs CNP Step 1	1.236	3.076	No	**ns**	-1.507 to 3.979
	BNP Step 1 vs CNP Step 1	0.8605	2.141	No	**ns**	-1.882 to 3.603
**Treatment at 38 days**	Ctr Step 2 vs ANP Step 2	-1044	52.15	Yes	***	-1181 to -907.3
	Ctr Step 2 vs BNP Step 2	-382.5	19.11	Yes	**	-519.1 to -245.8
	Ctr Step 2 vs CNP Step 2	-2.814	0.1406	No	**ns**	-139.4 to 133.8
	ANP Step 2 vs BNP Step 2	661.5	33.04	Yes	***	524.8 to 798.1
	ANP Step 2 vs CNP Step 2	1041	52.01	Yes	***	904.5 to 1178
	BNP Step 2 vs CNP Step 2	379.7	18.96	Yes	**	243.0 to 516.3
	**DAT 62kDa (di-glycosylated form)**					
	**Tukey's Multiple Comparison Test**	**Mean Diff.**	**q**	**Significant? P < 0.05?**	**Summary**	**95% CI of diff**
**Treatment at 31 days**	Ctr Step 1 vs ANP Step 1	-5663	58.19	Yes	***	-6327 to -4998
	Ctr Step 1 vs BNP Step 1	-3830	39.36	Yes	***	-4494 to -3166

	Ctr Step 1 vs CNP Step 1	-1430	14.7	Yes	**	-2095 to -766.1
	ANP Step 1 vs BNP Step 1	1833	18.83	Yes	**	1168 to 2497
	ANP Step 1 vs CNP Step 1	4232	43.49	Yes	***	3568 to 4897
	BNP Step 1 vs CNP Step 1	2400	24.66	Yes	**	1736 to 3064
**Treatment at 38 days**	Ctr Step 2 vs ANP Step 2	0.7327	1.947	No	**ns**	-1.835 to 3.301
	Ctr Step 2 vs BNP Step 2	0.7327	1.947	No	**ns**	-1.835 to 3.301
	Ctr Step 2 vs CNP Step 2	0.569	1.512	No	**ns**	-1.999 to 3.137
	ANP Step 2 vs BNP Step 2	0	0	No	**ns**	-2.568 to 2.568
	ANP Step 2 vs CNP Step 2	-0.1637	0.435	No	**ns**	-2.732 to 2.404
	BNP Step 2 vs CNP Step 2	-0.1637	0.435	No	**ns**	-2.732 to 2.404
	**DAT 75kDa (tri-glycosylated form)**					
	**Tukey's Multiple Comparison Test**	**Mean Diff.**	**q**	**Significant? P < 0.05?**	**Summary**	**95% CI of diff**
**Treatment at 31 days**	Ctr Step 1 vs ANP Step 1	-0.337	0.9777	No	**ns**	-2.690 to 2.016
	Ctr Step 1 vs BNP Step 1	-0.337	0.9777	No	**ns**	-2.690 to 2.016
	Ctr Step 1 vs CNP Step 1	0.1605	0.4656	No	**ns**	-2.192 to 2.513
	ANP Step 1 vs BNP Step 1	0	0	No	**ns**	-2.353 to 2.353
	ANP Step 1 vs CNP Step 1	0.4975	1.443	No	**ns**	-1.855 to 2.850
	BNP Step 1 vs CNP Step 1	0.4975	1.443	No	**ns**	-1.855 to 2.850
**Treatment at 38 days**	Ctr Step 2 vs ANP Step 2	-855.6	34.46	Yes	***	-1025 to -686.2
	Ctr Step 2 vs BNP Step 2	126.1	5.08	No	**ns**	-43.33 to 295.6
	Ctr Step 2 vs CNP Step 2	-356.1	14.34	Yes	**	-525.6 to -186.6
	ANP Step 2 vs BNP Step 2	981.8	39.54	Yes	***	812.3 to 1151
	ANP Step 2 vs CNP Step 2	499.5	20.12	Yes	**	330.1 to 669.0
	BNP Step 2 vs CNP Step 2	-482.2	19.42	Yes	**	-651.7 to -312.8

Fig 7C, D	*P<0.05; **P<0.01; ***P<0.001; ****P<0.0001	Cellular model: Human IPSCs from DONOR B				
	**β-catenin**					
	**Tukey's Multiple Comparison Test**	**Mean Diff.**	**q**	**Significant? P < 0.05?**	**Summary**	**95% CI of diff**
**Treatment at 31 days**	Ctr Step 1 vs ANP Step 1	-3.025	61.33	Yes	***	-3.362 to -2.689
	Ctr Step 1 vs BNP Step 1	-1.729	35.05	Yes	***	-2.066 to -1.392
	Ctr Step 1 vs CNP Step 1	-1.714	34.74	Yes	***	-2.050 to -1.377
	ANP Step 1 vs BNP Step 1	1.296	26.28	Yes	**	0.9597 to 1.633
	ANP Step 1 vs CNP Step 1	1.311	26.59	Yes	**	0.9748 to 1.648
	BNP Step 1 vs CNP Step 1	0.01515	0.3072	No	ns	-0.3215 to 0.3518
**Treatment at 38 days**	Ctr Step 2 vs ANP Step 2	-2.151	76.02	Yes	***	-2.345 to -1.958
	Ctr Step 2 vs BNP Step 2	-1.906	67.34	Yes	***	-2.099 to -1.713
	Ctr Step 2 vs CNP Step 2	-1.633	57.69	Yes	***	-1.826 to -1.439
	ANP Step 2 vs BNP Step 2	0.2457	8.681	Yes	*	0.05252 to 0.4388
	ANP Step 2 vs CNP Step 2	0.5188	18.33	Yes	**	0.3256 to 0.7119
	BNP Step 2 vs CNP Step 2	0.2731	9.65	Yes	*	0.07995 to 0.4663

	**Tyrosine hidroxylase (TH)**					
	**Tukey's Multiple Comparison Test**	**Mean Diff.**	**q**	**Significant? P < 0.05?**	**Summary**	**95% CI of diff**
**Treatment at 31 days**	Ctr Step 1 vs ANP Step 1	-0.1895	0.8951	No	ns	-1.635 to 1.256
	Ctr Step 1 vs BNP Step 1	-0.2502	1.182	No	ns	-1.695 to 1.195
	Ctr Step 1 vs CNP Step 1	-0.7745	3.658	No	ns	-2.220 to 0.6706
	ANP Step 1 vs BNP Step 1	-0.06072	0.2868	No	ns	-1.506 to 1.384
	ANP Step 1 vs CNP Step 1	-0.585	2.763	No	ns	-2.030 to 0.8601
	BNP Step 1 vs CNP Step 1	-0.5243	2.476	No	ns	-1.969 to 0.9209
**Treatment at 38 days**	Ctr Step 2 vs ANP Step 2	-0.1724	9.866	Yes	*	-0.2917 to -0.05313
	Ctr Step 2 vs BNP Step 2	-0.07365	4.215	No	ns	-0.1929 to 0.04561
	Ctr Step 2 vs CNP Step 2	-0.3074	17.59	Yes	**	-0.4267 to -0.1882
	ANP Step 2 vs BNP Step 2	0.09874	5.651	No	ns	-0.02052 to 0.2180
	ANP Step 2 vs CNP Step 2	-0.135	7.728	Yes	*	-0.2543 to -0.01579
	BNP Step 2 vs CNP Step 2	-0.2338	13.38	Yes	**	-0.3531 to -0.1145

	phospho TH					
	Tukey's Multiple Comparison Test	Mean Diff.	q	Significant? P < 0.05?	Summary	95% CI of diff
Treatment at 31 days	Ctr Step 1 vs ANP Step 1	-1.243	9.654	Yes	*	-2.122 to -0.3643
	Ctr Step 1 vs BNP Step 1	-1.164	9.038	Yes	*	-2.043 to -0.2850
	Ctr Step 1 vs CNP Step 1	-0.7569	5.878	No	ns	-1.636 to 0.1220
	ANP Step 1 vs BNP Step 1	0.07931	0.6159	No	ns	-0.7996 to 0.9582
	ANP Step 1 vs CNP Step 1	0.4863	3.776	No	ns	-0.3926 to 1.365
	BNP Step 1 vs CNP Step 1	0.407	3.16	No	ns	-0.4719 to 1.286
Treatment at 38 days	Ctr Step 2 vs ANP Step 2	0.4758	33.44	Yes	***	0.3787 to 0.5729
	Ctr Step 2 vs BNP Step 2	-0.07031	4.942	No	ns	-0.1674 to 0.02679
	Ctr Step 2 vs CNP Step 2	0.03206	2.254	No	ns	-0.06503 to 0.1292
	ANP Step 2 vs BNP Step 2	-0.5461	38.39	Yes	***	-0.6432 to -0.4490
	ANP Step 2 vs CNP Step 2	-0.4437	31.19	Yes	***	-0.5408 to -0.3466
	BNP Step 2 vs CNP Step 2	0.1024	7.196	Yes	*	0.005274 to 0.1995

	DAT 50kDa (not glycosylated form)					
	Tukey's Multiple Comparison Test	Mean Diff.	q	Significant? P < 0.05?	Summary	95% CI of diff
Treatment at 31 days	Ctr Step 1 vs ANP Step 1	-1019	86.47	Yes	***	-1099 to -938.1
	Ctr Step 1 vs BNP Step 1	-408.3	34.66	Yes	***	-488.7 to -327.9
	Ctr Step 1 vs CNP Step 1	-649.3	55.12	Yes	***	-729.7 to -568.9
	ANP Step 1 vs BNP Step 1	610.3	51.81	Yes	***	529.9 to 690.7
	ANP Step 1 vs CNP Step 1	369.2	31.34	Yes	***	288.8 to 449.6
	BNP Step 1 vs CNP Step 1	-241	20.46	Yes	**	-321.4 to -160.6
Treatment at 38 days	Ctr Step 2 vs ANP Step 2	-13658	86.9	Yes	***	-14731 to -12585
	Ctr Step 2 vs BNP Step 2	-9565	60.86	Yes	***	-10638 to -8492
	Ctr Step 2 vs CNP Step 2	-4212	26.8	Yes	***	-5285 to -3139
	ANP Step 2 vs BNP Step 2	4093	26.04	Yes	**	3020 to 5166
	ANP Step 2 vs CNP Step 2	9446	60.1	Yes	***	8373 to 10519
	BNP Step 2 vs CNP Step 2	5353	34.06	Yes	***	4280 to 6426

	DAT 58kDa (mono-glycosylated form)					
	**Tukey's Multiple Comparison Test**	**Mean Diff.**	**q**	**Significant? P < 0.05?**	**Summary**	**95% CI of diff**
**Treatment at 31 days**	Ctr Step 1 vs ANP Step 1	-760.1	49.23	Yes	***	-865.4 to -654.7
	Ctr Step 1 vs BNP Step 1	116.7	7.558	Yes	*	11.31 to 222.0
	Ctr Step 1 vs CNP Step 1	498.7	32.3	Yes	***	393.3 to 604.0
	ANP Step 1 vs BNP Step 1	876.7	56.79	Yes	***	771.4 to 982.1
	ANP Step 1 vs CNP Step 1	1259	81.53	Yes	***	1153 to 1364
	BNP Step 1 vs CNP Step 1	382	24.74	Yes	**	276.6 to 487.3
**Treatment at 38 days**	Ctr Step 2 vs ANP Step 2	-3094	76.55	Yes	***	-3370 to -2818
	Ctr Step 2 vs BNP Step 2	-827.2	20.47	Yes	**	-1103 to -551.3
	Ctr Step 2 vs CNP Step 2	-374.7	9.27	Yes	*	-650.5 to -98.82
	ANP Step 2 vs BNP Step 2	2267	56.09	Yes	***	1991 to 2543
	ANP Step 2 vs CNP Step 2	2719	67.28	Yes	***	2443 to 2995
	BNP Step 2 vs CNP Step 2	452.5	11.2	Yes	*	176.7 to 728.3
	DAT 62kDa (di-glycosylated form)					
	**Tukey's Multiple Comparison Test**	**Mean Diff.**	**q**	**Significant? P < 0.05?**	**Summary**	**95% CI of diff**
**Treatment at 31 days**	Ctr Step 1 vs ANP Step 1	-5816	46.94	Yes	***	-6529 to -5103
	Ctr Step 1 vs BNP Step 1	1392	11.24	Yes	**	678.8 to 2105
	Ctr Step 1 vs CNP Step 1	-2701	21.8	Yes	***	-3414 to -1987
	ANP Step 1 vs BNP Step 1	7208	58.18	Yes	***	6495 to 7921
	ANP Step 1 vs CNP Step 1	3115	25.14	Yes	***	2402 to 3828
	BNP Step 1 vs CNP Step 1	-4093	33.03	Yes	***	-4806 to -3379
**Treatment at 38 days**	Ctr Step 2 vs ANP Step 2	-1267	7.253	Yes	*	-2459 to -74.77
	Ctr Step 2 vs BNP Step 2	-12231	70.03	Yes	***	-13423 to -11039
	Ctr Step 2 vs CNP Step 2	562.8	3.222	No	ns	-629.2 to 1755
	ANP Step 2 vs BNP Step 2	-10964	62.78	Yes	***	-12156 to -9772
	ANP Step 2 vs CNP Step 2	1830	10.48	Yes	*	637.6 to 3022
	BNP Step 2 vs CNP Step 2	12794	73.25	Yes	***	11602 to 13986

	DAT 75kDa (tri-glycosylated form)					
	**Tukey's Multiple Comparison Test**	**Mean Diff.**	**q**	**Significant? P < 0.05?**	**Summary**	**95% CI of diff**
**Treatment at 31 days**	Ctr Step 1 vs ANP Step 1	-459.8	50.09	Yes	***	-522.4 to -397.1
	Ctr Step 1 vs BNP Step 1	78.58	8.561	Yes	*	15.94 to 141.2
	Ctr Step 1 vs CNP Step 1	-79.47	8.659	Yes	*	-142.1 to -16.83
	ANP Step 1 vs BNP Step 1	538.4	58.66	Yes	***	475.7 to 601.0
	ANP Step 1 vs CNP Step 1	380.3	41.44	Yes	***	317.7 to 442.9
	BNP Step 1 vs CNP Step 1	-158	17.22	Yes	**	-220.7 to -95.41
**Treatment at 38 days**	Ctr Step 2 vs ANP Step 2	-325.1	62.36	Yes	***	-355.2 to -295.1
	Ctr Step 2 vs BNP Step 2	-112.7	21.61	Yes	***	-142.7 to -82.67
	Ctr Step 2 vs CNP Step 2	-2.022	0.3878	No	ns	-32.04 to 27.99
	ANP Step 2 vs BNP Step 2	212.5	40.75	Yes	***	182.4 to 242.5
	ANP Step 2 vs CNP Step 2	323.1	61.97	Yes	***	293.1 to 353.1
	BNP Step 2 vs CNP Step 2	110.7	21.23	Yes	***	80.65 to 140.7
