# Supplementary figures and images for: Natriuretic peptides are neuroprotective on in vitro models of PD and promote dopaminergic differentiation of hiPSCs-derived neurons via the Wnt/β-catenin signaling
Source: Cell Death Discov. 2021 Nov 1;7:330. doi: 10.1038/s41420-021-00723-6 (PMC8560781; doi:10.1038/s41420-021-00723-6)

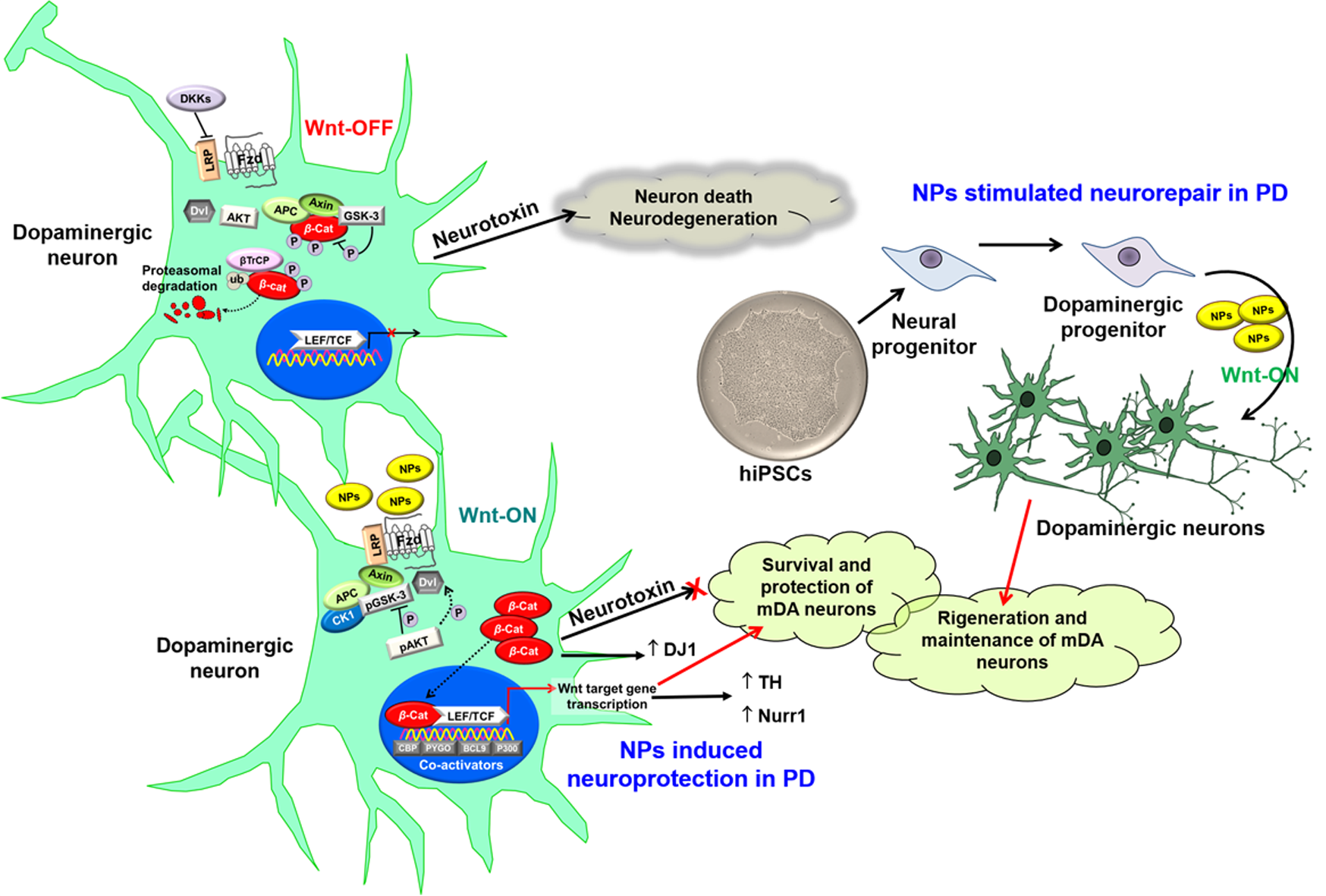

Supplement: Supplementary file 5 — Graphical Abstract [file 41420_2021_723_MOESM5_ESM.tif]
